# Inactivation of VCP/ter94 Suppresses Retinal Pathology Caused by Misfolded Rhodopsin in *Drosophila*

**DOI:** 10.1371/journal.pgen.1001075

**Published:** 2010-08-26

**Authors:** Ana Griciuc, Liviu Aron, Michel J. Roux, Rüdiger Klein, Angela Giangrande, Marius Ueffing

**Affiliations:** 1 Department of Protein Science, Helmholtz Zentrum Muenchen-German Research Center for Environmental Health, Neuherberg, Germany; 2 Department of Molecular Neurobiology, Max-Planck-Institute of Neurobiology, Martinsried, Germany; 3 Department of Neurobiology and Genetics, Institut de Génétique et de Biologie Moléculaire et Cellulaire, CNRS/INSERM/ULP, Illkirch, France; 4 Department of Cell and Developmental Biology, Institut de Génétique et de Biologie Moléculaire et Cellulaire, CNRS/INSERM/ULP, Illkirch, France; 5 Institute for Ophthalmic Research, Center for Ophthalmology, University of Tuebingen, Tuebingen, Germany; University of Texas Health Science Center, United States of America

## Abstract

The most common Rhodopsin (Rh) mutation associated with autosomal dominant *retinitis pigmentosa* (ADRP) in North America is the substitution of proline 23 by histidine (Rh^P23H^). Unlike the wild-type Rh, mutant Rh^P23H^ exhibits folding defects and forms intracellular aggregates. The mechanisms responsible for the recognition and clearance of misfolded Rh^P23H^ and their relevance to photoreceptor neuron (PN) degeneration are poorly understood. Folding-deficient membrane proteins are subjected to Endoplasmic Reticulum (ER) quality control, and we have recently shown that Rh^P23H^ is a substrate of the ER–associated degradation (ERAD) effector VCP/ter94, a chaperone that extracts misfolded proteins from the ER (a process called retrotranslocation) and facilitates their proteasomal degradation. Here, we used *Drosophila*, in which Rh1^P37H^ (the equivalent of mammalian Rh^P23H^) is expressed in PNs, and found that the endogenous Rh1 is required for *Rh1^P37H^* toxicity. Genetic inactivation of *VCP* increased the levels of misfolded Rh1^P37H^ and further activated the Ire1/Xbp1 ER stress pathway in the *Rh1^P37H^* retina. Despite this, *Rh1^P37H^* flies with decreased *VCP* function displayed a potent suppression of retinal degeneration and blindness, indicating that VCP activity promotes neurodegeneration in the *Rh1^P37H^* retina. Pharmacological treatment of *Rh1^P37H^* flies with the VCP/ERAD inhibitor Eeyarestatin I or with the proteasome inhibitor MG132 also led to a strong suppression of retinal degeneration. Collectively, our findings raise the possibility that excessive retrotranslocation and/or degradation of visual pigment is a primary cause of PN degeneration.

## Author Summary

Patients affected by autosomal dominant *retinitis pigmentosa* (ADRP) experience gradual loss of vision, and mutations in the visual pigment Rhodopsin—a G protein-coupled receptor that mediates phototransduction—are associated with ADRP. The most common ADRP mutation is the substitution of proline 23 by histidine (Rh^P23H^), which causes Rh misfolding and aggregation. It is currently unclear how mutant Rh^P23H^ leads to photoreceptor neuron (PN) degeneration in ADRP. We used the fruitfly *Drosophila melanogaster* in which Rh1^P37H^ (the equivalent of mammalian Rh^P23H^) was overexpressed in PNs. We found that the presence of both mutant Rh1^P37H^ and endogenous Rh1 is required for neurodegeneration in *Rh1^P37H^* flies. To understand the impact of Rh1 misfolding and clearance on PN degeneration, we inactivated the chaperone VCP/ter94, which escorts misfolded proteins out of the ER (a process termed retrotranslocation) and delivers them for proteasomal degradation. *Rh1^P37H^* flies with decreased *VCP* function displayed more misfolded Rh1^P37H^ but, remarkably, showed a potent suppression of PN degeneration and blindness. Treatment of *Rh1^P37H^* flies with VCP or proteasome inhibitors also mitigated PN degeneration. Our results suggest that excessive retrotranslocation and/or degradation of visual pigment is deleterious for PN expressing misfolded Rh^P23H^.

## Introduction

Collapse of protein homeostasis (proteostasis) due to protein misfolding and aggregation is the central pathogenic event in neurodegenerative disease [1]. *Retinitis pigmentosa* (RP) represents a group of disorders characterized by progressive loss of photoreceptor neurons (PNs) and blindness [2]–[4], and mutations in the visual pigment Rhodopsin (Rh) are the most prevalent genetic defects causing RP [5], [6]. Rh is a G protein-coupled receptor that initiates the phototransduction cascade and more than 120 Rh point mutations have been associated with RP; most of these Rh mutations act dominantly to cause autosomal dominant RP (ADRP), while some mutations cause recessive RP [7]. Based on their biochemical and cellular properties, Rh mutations have been grouped into several classes [7]. Substitution of proline 23 by histidine (Rh^P23H^) is the most common genetic defect associated with ADRP in North America and is arguably the best characterized Rh mutation to date [3], [7], [8]. Unlike wild-type (WT) Rh, which is properly delivered to the plasma membrane, mutant Rh^P23H^ fails to fold properly [3], [5], [9], [10], appears to exhibit enhanced retention within the endoplasmic reticulum (ER) [3], [5], [7], [11]–[13] and forms intracellular aggregates [14]–[16].

The presence of misfolded proteins in the ER activates the unfolded protein response (UPR), an adaptive process which helps reduce the load of unfolded proteins; chronic and excessive UPR activation might be pro-apoptotic [17]–[19], although a moderate increase in UPR activation (via the Ire1/Xbp1 pathway) is protective for PNs [20]. In order to reduce protein misfolding-induced stress within the ER, proteins with folding defects are identified and cleared during a process called ER-associated degradation (ERAD), which involves their export from the ER to the cytosol (retrotranslocation) and degradation by the proteasome [21], [22]. The ATP-dependent chaperone Valosin-containing protein VCP/ter94/p97/cdc48 is the driving force for the retrotranslocation of misfolded proteins [23]–[25]. Two ATPase domains (D1 and D2) generate the energy necessary for VCP to promote extraction of misfolded substrates from the ER and delivery to the proteasome [26], [27]. Increased ERAD/VCP activity leads to degradation of the cystic fibrosis-linked mutant ΔF508-CFTR, which retains some functionality despite being misfolded [28]. VCP also promotes degradation of the human V2 vasopressin receptor in X-linked nephrogenic diabetes insipidus [29]. VCP co-localizes with aggregated Ataxin-3 [30], TDP-43 [31] or with ubiquitinated inclusions in Alzheimer's and Parkinson's disease [32] suggesting that modulation of VCP activity might have a broad relevance for protein clearance (including neurodegenerative) disorders.

The mechanisms linking mutant Rh^P23H^ to PN degeneration in ADRP are incompletely understood. Rh^P23H^ and other misfolded Rh mutants were found to recruit the endogenous WT Rh into aggregates (dominant-negative [DN] effect) leading to decreased levels of mature WT Rh [15], [33]–[36]. The mutant Rh^P23H^ might acquire novel properties (gain-of-function [GOF] mechanism) such as: aggregate formation or inclusion formation in the cytosol; generation of ER stress and activation of the UPR pathways; other toxic effects on diverse cellular processes [7], [11], [15], [18], [37]. Therefore, a better understanding of the cellular and molecular mechanisms mediating dominance in *Rh^P23H^*-linked RP is required for the development of effective therapies [7].

We have recently shown that misfolded Rh^P23H^ forms a complex with the ERAD effector VCP in mammalian cell culture systems. The interaction is maximal when both the N-terminal and the D1 ATPase domains of VCP are present; furthermore, VCP uses its D2 ATPase domain to promote Rh^P23H^ retrotranslocation and proteasomal delivery [16]. It remained unclear, however, how the activity of VCP impacts on the *Rh^P23H^*-mediated retinal degeneration and blindness.

To address this question, we have used the previously established *Drosophila* model of *Rh^P23H^*-linked RP, in which overexpression of Rh1^P37H^ (the equivalent of mammalian Rh^P23H^) in photoreceptor neurons leads to age- and light-dependent retinal degeneration and blindness [11]. Here, we show that the degeneration of PNs requires the presence of both mutant Rh1^P37H^ and endogenous WT Rh1. Genetic inactivation of the ERAD retrotranslocator *VCP* increased the levels of misfolded Rh1^P37H^ but surprisingly, conferred protection against *Rh1^P37H^*-mediated retinal degeneration. Pharmacological inactivation of VCP/ERAD or proteasome activities also led to a robust suppression of *Rh1^P37H^*-mediated neurodegeneration. Our results suggest that excessive retrotranslocation and/or proteasomal degradation of visual pigment is pathogenic for PNs.

## Results

### Proteostasis Defects and Retinal Degeneration in *Rh1^P37H^*-Expressing Flies

**Figure 1:**
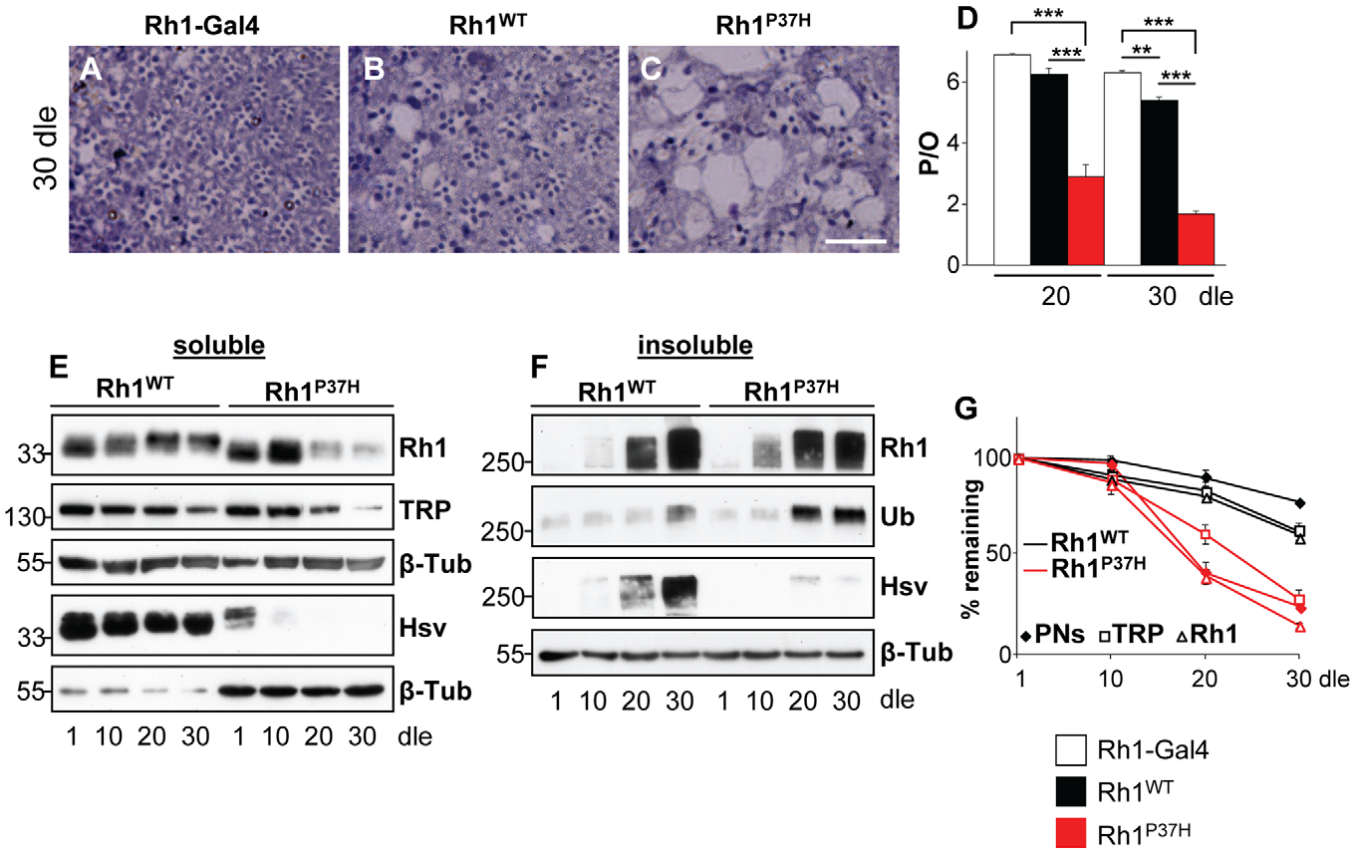
Progressive retinal degeneration and Rh1 loss in *Rh1^P37H^*-expressing flies exposed to light. (A–C) Photomicrographs of toluidine blue stained semithin eye sections of *Rh1-Gal4* (A), *Rh1^WT^;Rh1^+/+^* (B) and *Rh1^P37H^;Rh1^+/+^* (C) flies kept in light for 30 days. Dle: days of light exposure. Scale bar is 50 µm. (D) Quantification of average number of photoreceptors/ommatidium (P/O) in different mutant groups (n>7 animals in each group, **p<0.01 and *** p<0.001 t-test). *Rh1^P37H^;Rh1^+/+^* flies display a dramatic loss of P/O after 20 dle. (E,F) Immunoblots revealing the abundance of total (endogenous and ectopic, Rh1 antibody) and ectopic (hsv antibody) Rh1 in detergent-soluble (E) or insoluble (F) fractions obtained from retinas of *Rh1^WT^;Rh1^+/+^* or *Rh1^P37H^;Rh1^+/+^* flies exposed to light for the indicated durations. The rhabdomeric marker TRP was used to independently assess rhabdomere loss (E) while aggregates were independently labeled with an Ubiquitin antibody (F). β-Tubulin (β-Tub) served as loading control. Please note that 5-fold less protein (E) was loaded for *Rh1^WT^;Rh1^+/+^* flies for the hsv WB. (G) Graph describing the changes of mature Rh1 levels (triangles), TRP levels (squares) and retinal degeneration (P/O; rhombus) in *Rh1^WT^;Rh1^+/+^* and *Rh1^P37H^;Rh1^+/+^* flies exposed to light for increasing durations.

To understand how targeting and clearance of misfolded Rh impact on the maintenance of PNs in RP, we used a previously established *Drosophila* RP model, in which mutant Rh1^P37H^ (the equivalent of mammalian Rh^P23H^) is ectopically expressed in PNs R1-6, under the control of a promoter identical to the endogenous *Rh1* promoter [11]. Flies overexpressing mutant *Rh1^P37H^* (genotype: *Rh1^P37H^;Rh1^+/+^*), in contrast to flies overexpressing WT *Rh1* (genotype: *Rh1^WT^;Rh1^+/+^*) and to control (*Rh1-Gal4*) flies display a dramatic loss of PNs after 20 and 30 days of light exposure (dle) (Figure 1A–1D). After 30 dle, ommatidia from *Rh1^P37H^;Rh1^+/+^* retinas displayed 1.7 photoreceptors on average, compared to 5.5 in *Rh1^WT^;Rh1^+/+^* and 6.5 in *Rh1-Gal4* flies (n>7 eyes/genotype and at least 150 ommatidia were analyzed/eye; ** p<0.01 and *** p<0.001 student's t-test). No degeneration was seen in *Rh1^P37H^;Rh1^+/+^* flies reared at light at postnatal day 1 (P1) or reared in the dark at P30 (Figure S1). Thus, *Rh1^P37H^* induces age- and light-dependent retinal degeneration in *Drosophila* PNs.

In order to characterize the misfolded Rh1-induced proteostasis defects in our transgenic flies, we collected fly heads of defined genotypes and ages and following lysis in detergent-containing RIPA modified buffer (see Materials and Methods), we separated the lysates into detergent-soluble and insoluble fractions. We expected most of the mature (rhabdomeric) Rh1 to be contained within the detergent-soluble fractions, while aggregation-prone Rh1 should be enriched in the detergent-insoluble fraction [14], [15]. To specifically detect the overexpressed protein, we used flies in which ectopically expressed Rh1^P37H^ (or Rh1^WT^) was hsv-tagged [11], [35]; in the following, all *Rh1^P37H-hsv^;Rh1^+/+^* and *Rh1^WT-hsv^;Rh1^+/+^* flies are named *Rh1^P37H^;Rh1^+/+^* and *Rh1^WT^;Rh1^+/+^* respectively, unless otherwise stated. When analyzing detergent-soluble fractions, we found that 1-day-old *Rh1^WT^;Rh1^+/+^* flies displayed 55±7% more Rh1 than *Rh1-Gal4* flies, both when reared in the dark or at light (Figure S2A and Figure S3A and S3B) indicating that the *Rh1* promoter induces a moderate transgene expression (also seen by [11]). Similar to *Rh1^WT^*-expressing flies, 1-day-old *Rh1^P37H^;Rh1^+/+^* flies displayed 51±8% more Rh1 than *Rh1-Gal4* flies when reared in the dark (Figure S3B); interestingly, the same flies reared at light displayed only 9±1% more Rh1 than *Rh1-Gal4* flies (Figure S3A). Moreover, light exposure (in contrast to dark rearing) led to loss of the hsv signal in *Rh1^P37H^;Rh1^+/+^* but not *Rh1^WT^;Rh1^+/+^* flies (Figure S3C), suggesting that light exposure exacerbates the Rh1^P37H^ maturation defects. We also evaluated *Rh1^P37H^* transgene expression on a *Rh1* null background. Flies expressing one copy of the *Rh1^P37H^* transgene on a complete *Rh1* null background (*Rh1^P37H^;Rh1^−/−^*), or two copies of the mutant transgene on a complete *Rh1* null background (*Rh1^P37H^/Rh1^P37H^;Rh1^−/−^*) had significantly higher levels of mature Rh1^P37H^ when reared at light; Rh1 labeling revealed that *Rh1^P37H^/Rh1^P37H^;Rh1^−/−^* flies (in which all Rh1 is Rh1^P37H^) exhibited levels of mature Rh1^P37H^ that were comparable to those of the endogenous Rh1 in *Rh1^P37H^;Rh1^+/+^* flies (Figure S3D), reflecting the increased dosage of the Rh1^P37H^ transgene. This suggests that the mutant Rh1^P37H^ is able to traffic through the secretory pathway and this process is impaired by light exposure.

We then performed a time-course analysis of Rh1 levels in our transgenic and control flies exposed to light and found a dramatic loss of mature Rh1 (endogenous and ectopic) in *Rh1^P37H^;Rh1^+/+^* but not *Rh1^WT^;Rh1^+/+^* flies after 20 dle (Figure 1E). We used an hsv-specific antibody to label the ectopic protein and found a complete loss of hsv signal in the *Rh1^P37H^;Rh1^+/+^* soluble fraction after 10 dle, while no loss of mature Rh1 occurred in *Rh1^WT^;Rh1^+/+^* flies. As mentioned above, this loss of hsv signal might be due to Rh1^P37H^ maturation defects, and/or to excessive Rh1^P37H^ degradation. We next assessed the situation of Rh1-containing species in the detergent-insoluble fraction. We found a significant increase in Rh1-containing oligomeric species that migrated at approximately 250–300 kDa, in both *Rh1^WT^;Rh1^+/+^* and *Rh1^P37H^;Rh1^+/+^* flies after 20 dle (Figure 1F; whole WB scans can be seen in Figure S4). These species represent small Rh1 oligomers (probably containing 6–10 Rh1 molecules) that were extracted using our detergent treatment protocol (and supposedly found as such *in vivo*), although they might also have assembled *ex-vivo* [14], [38]. Control (*Rh1-Gal4*) retinas displayed steady levels of mature Rh1 in the detergent-soluble fraction, while some traces of oligomeric Rh1 were detected in the detergent-insoluble fraction (Figure S2). Since Rh^P23H^-containing oligomeric species were previously found to be ubiquitinated [14], [36], we used an Ubiquitin-specific antibody to label Rh1-containing oligomeric species in *Drosophila*, and found a robust signal after 20 dle in *Rh1^P37H^;Rh1^+/+^* flies (Figure 1F). This suggests that Rh1-containing oligomers are ubiquitinated, although it remains possible that other (Rh1-negative) species are also Ubiquitin-positive. Remarkably, hsv labeling was absent in the insoluble fraction from *Rh1^P37H^;Rh1^+/+^*, but not *Rh1^WT^;Rh1^+/+^* flies, raising the possibility that endogenous Rh1 is the major component of these Rh1 oligomeric species from the *Rh1^P37H^* retina (Figure 1F). It remains, however, possible that the lack of hsv signal is due to epitope unavailability (due to oligomerization) or that the hsv tag was cleaved. Accumulation of endogenous Rh1 in the insoluble fraction from the *Rh1^P37H^;Rh1^+/+^* retina might be a consequence of WT Rh1 recruitment by the mutant Rh1^P37H^ [15], [33]–[36]. To determine whether the loss of mature Rh1 is the cause or the consequence of retinal degeneration in *Rh1^P37H^;Rh1^+/+^* flies, we compared the time-dependent decrease in mature (rhabdomeric) Rh1 levels, the decrease of the rhabdomeric marker TRP [39] levels and the percentage of surviving PNs in the *Rh1^P37H^;Rh1^+/+^* retina (Figure 1G; results from 3 independent crosses were averaged). As expected, death of PNs parallels the decrease in TRP levels, consistent with the rhabdomeres being lost during retinal degeneration; moreover, we found that no loss of mature Rh1 precedes the loss of PNs/TRP (Figure 1G). Therefore, the onset of retinal degeneration in *Rh1^P37H^*-expressing flies is not caused by loss of mature Rh1; the reverse is true, i.e. loss of mature Rh1 is the result of rhabdomere loss, which takes place during retinal degeneration. We also used INAD as an independent rhabdomeric marker and found a similar profile to TRP (data not shown). Furthermore, given the similar amounts of Rh1 oligomers in *Rh1^WT^;Rh1^+/+^* and *Rh1^P37H^;Rh1^+/+^* flies – despite marked differences in retinal integrity – the formation of Rh1 oligomers is probably not a primary cause of retinal degeneration in *Rh1^P37H^;Rh1^+/+^* flies.

### The Endogenous *Rh1* Is Required for *Rh1^P37H^* Toxicity

**Figure 2:**
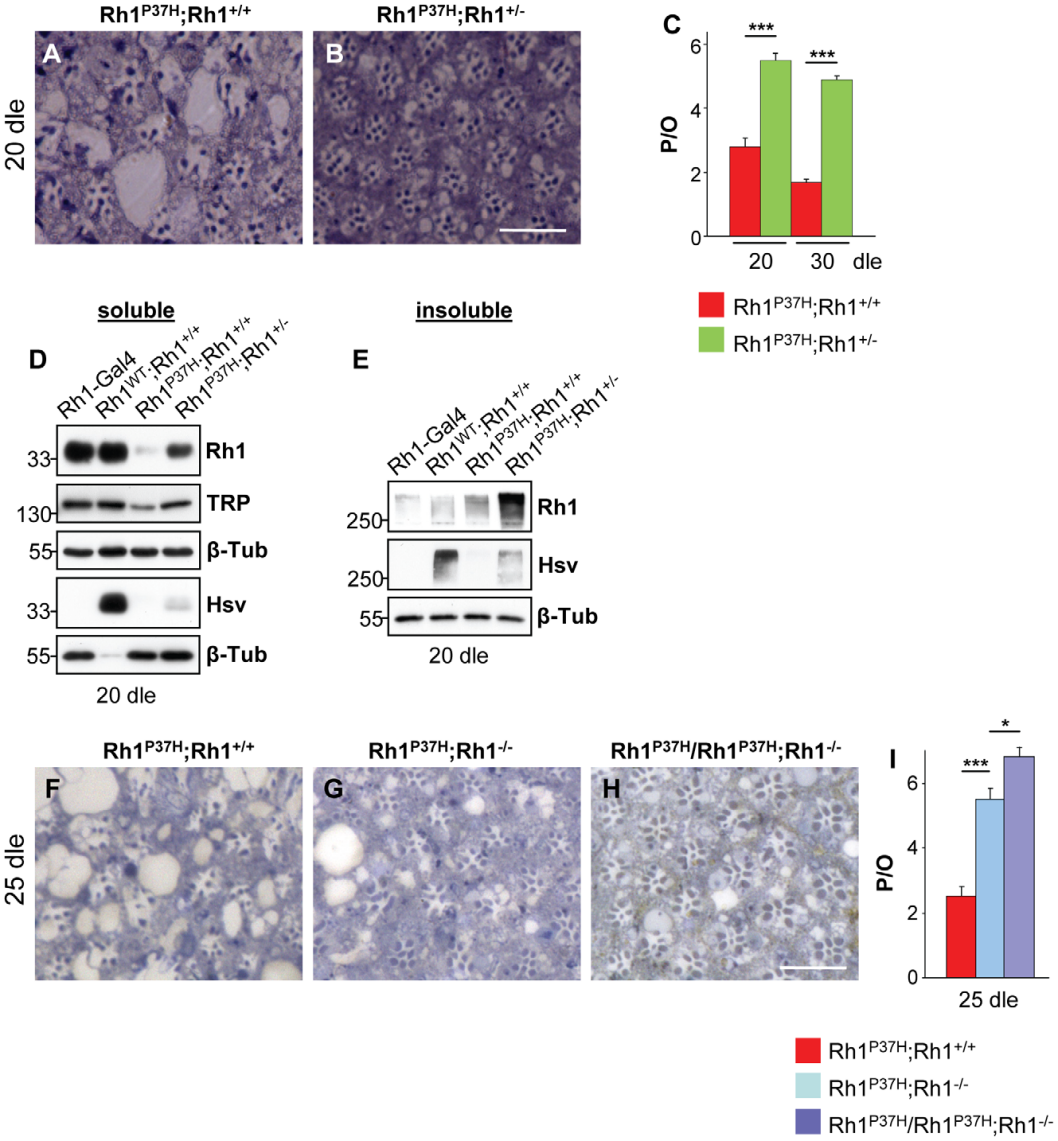
The endogenous Rh1 is required for *Rh1^P37H^* toxicity. (A–C,F–I) Reducing the dosage of endogenous *Rh1* rescues retinal degeneration in *Rh1^P37H^;Rh1^+/+^* flies, suggesting that endogenous Rh1 is required for *Rh1^P37H^* toxicity. Photomicrographs of toluidine blue sections of *Rh1^P37H^;Rh1^+/+^* (A,F), *Rh1^P37H^;Rh1^+/−^*(B), *Rh1^P37H^;Rh1^−/−^* (G) and *Rh1^P37H^/Rh1^P37H^;Rh1^−/−^* (H) flies at 20 dle (A,B) and 25 dle (F–H) and (C,I) quantification of the average number of P/O (n>7 animals/group, * p<0.05 and *** p<0.001 t-test). Scale bar is 50 µm. (D,E) Immunoblots showing the levels of total and ectopic mature (D) and aggregated Rh1 (E), as well as TRP levels in flies of indicated genotypes, after 20 dle. β-Tubulin (β-Tub) served as loading control. Overexpressed WT or P37H Rh1 was hsv-tagged and *Rh1-Gal4* flies, lacking hsv, served as negative control for the hsv antibody Please note that 10-fold less protein (D) was loaded for *Rh1^WT^;Rh1^+/+^* flies for the hsv WB.

The recruitment of endogenous Rh1 into insoluble aggregates prompted us to investigate the relevance of endogenous Rh1 to the retinal pathology initiated by Rh1^P37H^. To reduce the dosage of the endogenous *Rh1* in *Rh1^P37H^;Rh1^+/+^* flies, we used the *Rh1* null allele *ninaE^I17^* (here called *Rh1^−^*). We found, remarkably, that PN degeneration was strongly suppressed in *Rh1^P37H^;Rh1^+/−^* flies (Figure 2A–2C; n>7 eyes/genotype), suggesting that the endogenous Rh1 is required for *Rh1^P37H^* toxicity. Consistent with the rescue of PN degeneration, the levels of TRP and of total mature Rh1, but not P37H ectopic Rh1 (hsv blot), were partially (51±5% and 59±6,5% respectively) restored (Figure 2D), indicating that rescue of retinal degeneration in *Rh1^P37H^;Rh1^+/−^* flies correlated with decreased degradation of endogenous Rh1. Interestingly, increased dosage of the mutant *Rh1^P37H^* transgene relative to endogenous *Rh1* led to an increased level of Rh1 oligomers in the detergent-insoluble fraction (that contained a large fraction of endogenous Rh1; Figure 2E, compare Rh1 and hsv blots) in *Rh1^P37H^;Rh1^+/−^* flies. We then wondered whether the mutant Rh1^P37H^ is toxic by itself, i.e. if the presence of Rh1^P37H^ in PNs completely deprived of endogenous Rh1 is sufficient to cause a strong PN degeneration. For this, we generated *Rh1^P37H^;Rh1^−/−^* flies (carrying one copy of the *Rh1^P37H^* transgene on a *Rh1* null background) and *Rh1^P37H^*/*Rh1^P37H^;Rh1^−/−^* flies (carrying two copies of the *Rh1^P37H^* transgene on a *Rh1* null background). As mentioned above, Rh1^P37H^ maturation was significantly improved in these mutants, (Figure S3D). When analyzing the retinal integrity after 25 dle, we found, remarkably, that PN degeneration was largely suppressed in *Rh1^P37H^;Rh1^−/−^* and *Rh1^P37H^*/*Rh1^P37H^;Rh1^−/−^* flies (Figure 2F–2I; n = 7 eyes/genotype). Analysis of Rh1 levels at 25 dle also confirmed the rescue of retinal degeneration (Figure S5). Therefore, the *Rh1^P37H^* allele is not toxic by itself, as it is insufficient to cause retinal degeneration when expressed in the absence of the endogenous Rh1. Retinal degeneration in *Rh1^P37H^*;*Rh1^+/+^* flies therefore depends on a cellular environment containing both misfolded Rh1^P37H^ and endogenous WT Rh1.

### VCP Is Required *In Vivo* for Clearance of Misfolded Rh1^P37H^

**Figure 3:**
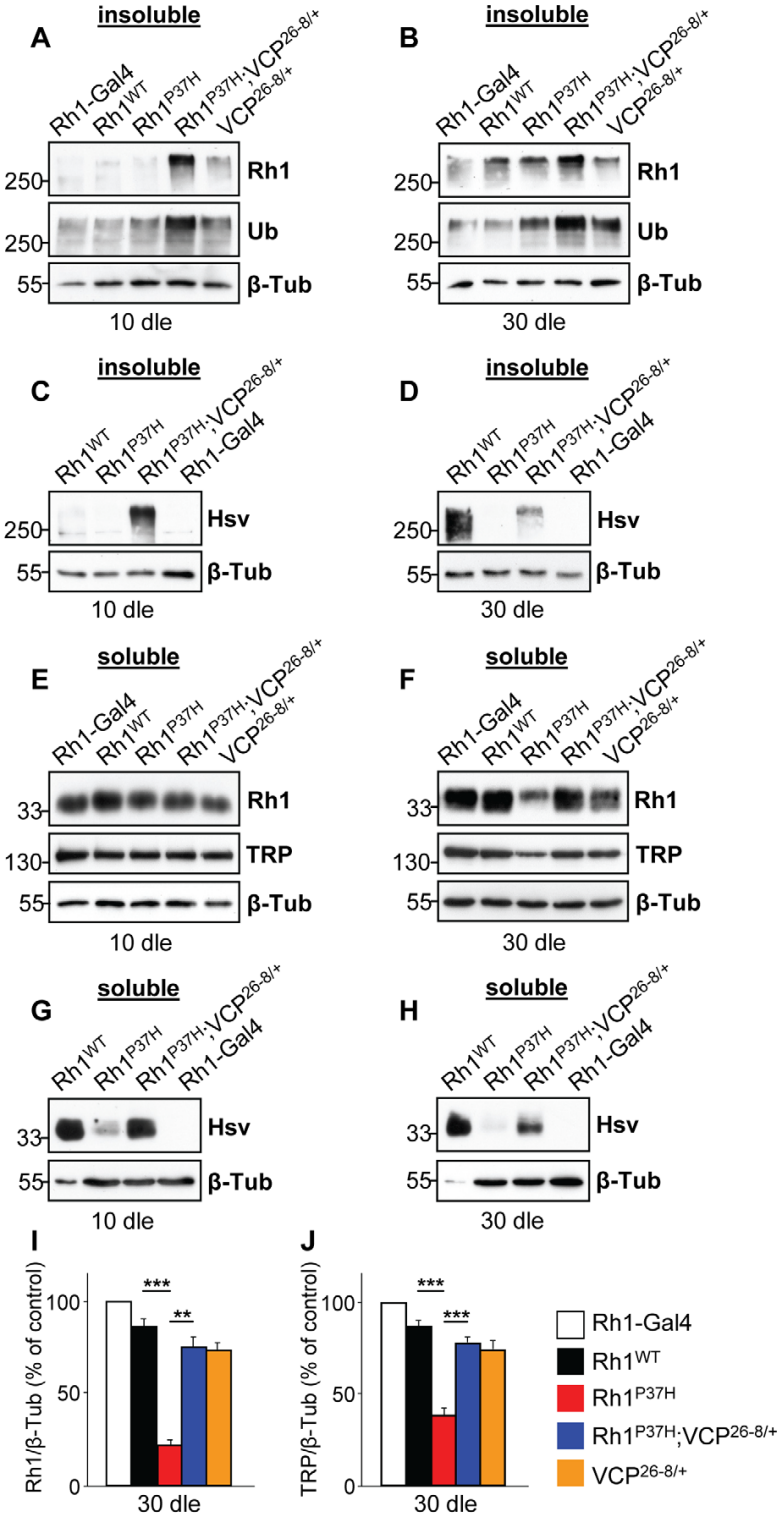
Decreasing *VCP* function increases Rh1 aggregate load and restores mature Rh1 levels in *Rh1^P37H^*-expressing flies. (A–D) Immunoblots showing the levels of total (A,B, endogenous and ectopic, Rh1 antibody) and ectopic (C,D, hsv antibody) Rh1 aggregates in flies of indicated genotypes after 10 (dle) (A,C) or 30 (B,D) days of light exposure (dle). An Ubiquitin-specific antibody was used to independently label aggregates. *Rh1^P37H^;VCP^26-8/+^;Rh1^+/+^* flies display increased aggregate levels compared to the other mutant flies. (E–H) Immunoblots revealing the levels of total (E,F, endogenous and ectopic, Rh1 antibody) and ectopic (G,H, hsv antibody) mature Rh1, as well as TRP levels in flies of indicated genotypes after 10 dle (E,G) and 30 (F,H) dle. Loss of endogenous and ectopic mature Rh1 in *Rh1^P37H^;Rh1^+/+^* flies is rescued in *Rh1^P37H^;VCP^26-8/+^;Rh1^+/+^* flies. Overexpressed WT or P37H Rh1 was hsv-tagged and *Rh1-Gal4* flies, lacking hsv, served as negative control for the hsv antibody. Note that 10-fold less protein was loaded for the *Rh1^WT^;Rh1^+/+^* flies (G,H) and β-Tubulin (β-Tub) served as loading control. (I,J) Quantifications of Rh1 levels (I) and of TRP levels (J) in flies of indicated genotypes, after 30 dle. Values from three independent experiments were averaged (**p<0.01 and *** p<0.001 t-test).

To investigate the relevance of Rh1^P37H^ retrotranslocation and clearance to retinal degeneration, we focused our attention on the ERAD effector VCP/ter94/p97/cdc48, the driving force for extraction of misfolded proteins from the ER and delivery to the proteasome [23], [24], [27]. To test whether VCP activity is required for clearance of misfolded Rh1, we reduced *VCP* function using the hypomorphic allele *ter94^26-8^* [40] that we refer to as *VCP^26-8^* (Figure S6A). Using Rh1- and Ubiquitin-specific antibodies to label oligomeric species, we found that *Rh1^P37H^;VCP^26-8/+^;Rh1^+/+^* flies, in contrast to *Rh1^P37H^;Rh1^+/+^* and control flies, displayed a strong increase in total levels of oligomeric Rh1 at 10 dle (Figure 3A) and similarly at 20 (Figure S6B) and 30 dle (Figure 3B). Flies carrying the *VCP^26-8^* allele in an otherwise *WT* background (genotype: *VCP^26-8/+^;Rh1^+/+^*) also displayed slightly more oligomeric Rh1 relative to control flies (Figure 3A and 3B) suggesting that VCP is involved in the quality control of endogenous Rh1, which might be a client of ERAD under physiological conditions [41]. When using hsv antibody to label the ectopic Rh1^P37H^, we found an increased signal in the *Rh1^P37H^;VCP^26-8/+^;Rh1^+/+^* retina as compared to the *Rh1^P37H^;Rh1^+/+^* retina (Figure 3C and 3D and Figure S6C). Thus, partial *VCP* inactivation prevents the loss of misfolded Rh1^P37H^. Importantly, this finding also indicates that we are able to detect oligomeric species containing the mutant Rh1^P37H^. Therefore, the absence of Rh1^P37H^ from the insoluble fractions of *Rh1^P37H^;Rh1^+/+^* flies (Figure 1F) suggests that the mutant Rh1^P37H^ was degraded, in a process that required VCP activity. Since the increase in Rh1-containing oligomeric species was observed before the onset of retinal degeneration (at 10 dle), we can rule out that this effect was indirectly caused by differences in retinal integrity. Thus, VCP activity is required *in vivo* for degradation of misfolded Rh1^P37H^.

To determine the levels of mature (soluble) Rh1 in retinas derived from *Rh1^P37H^;VCP^26-8/+^;Rh1^+/+^, Rh1^P37H^;Rh1^+/+^* or control flies, we used detergent-soluble fractions and a Rh1-specific antibody to reveal their content of mature Rh1. While the levels of mature total (endogenous and ectopic) Rh1 were similar for all groups after 10 dle (Figure 3E), we found a dramatic loss of mature Rh1 in *Rh1^P37H^;Rh1^+/+^* flies after 20 dle (Figure S6D) and 30 dle (Figure 3F and 3I; see also Figure 1E). Remarkably, partial *VCP* inactivation led to an almost complete rescue of mature Rh1 levels (Figure 3F and 3I; results from 3 independent crosses were averaged) and similarly, restored the levels of the rhabdomeric marker TRP (Figure 3F and 3J), suggesting decreased retinal degeneration in the *Rh1^P37H^;VCP^26-8/+^;Rh1^+/+^* retina after 30 dle. We observed a similar, although moderate, rescue of the mature ectopic Rh1^P37H^ (hsv-labeled) after 20 dle (Figure S6E) and 30 dle (Figure 3H), but also after 10 dle (Figure 3G) and 1 dle (data not shown). The increased levels of mature Rh1^P37H^ in the intact (1–10 dle) *Rh1^P37H^;VCP^26-8/+^;Rh1^+/+^* retina suggests that partial *VCP* inactivation not only prevents Rh1^P37H^ degradation but also allows some of the mutant Rh1^P37H^ to traffic through the secretory pathway.

### Increased Ire1/Xbp1 Pathway Activation in *Rh1^P37H^*-Expressing Flies with Decreased *VCP* Function

**Figure 4:**
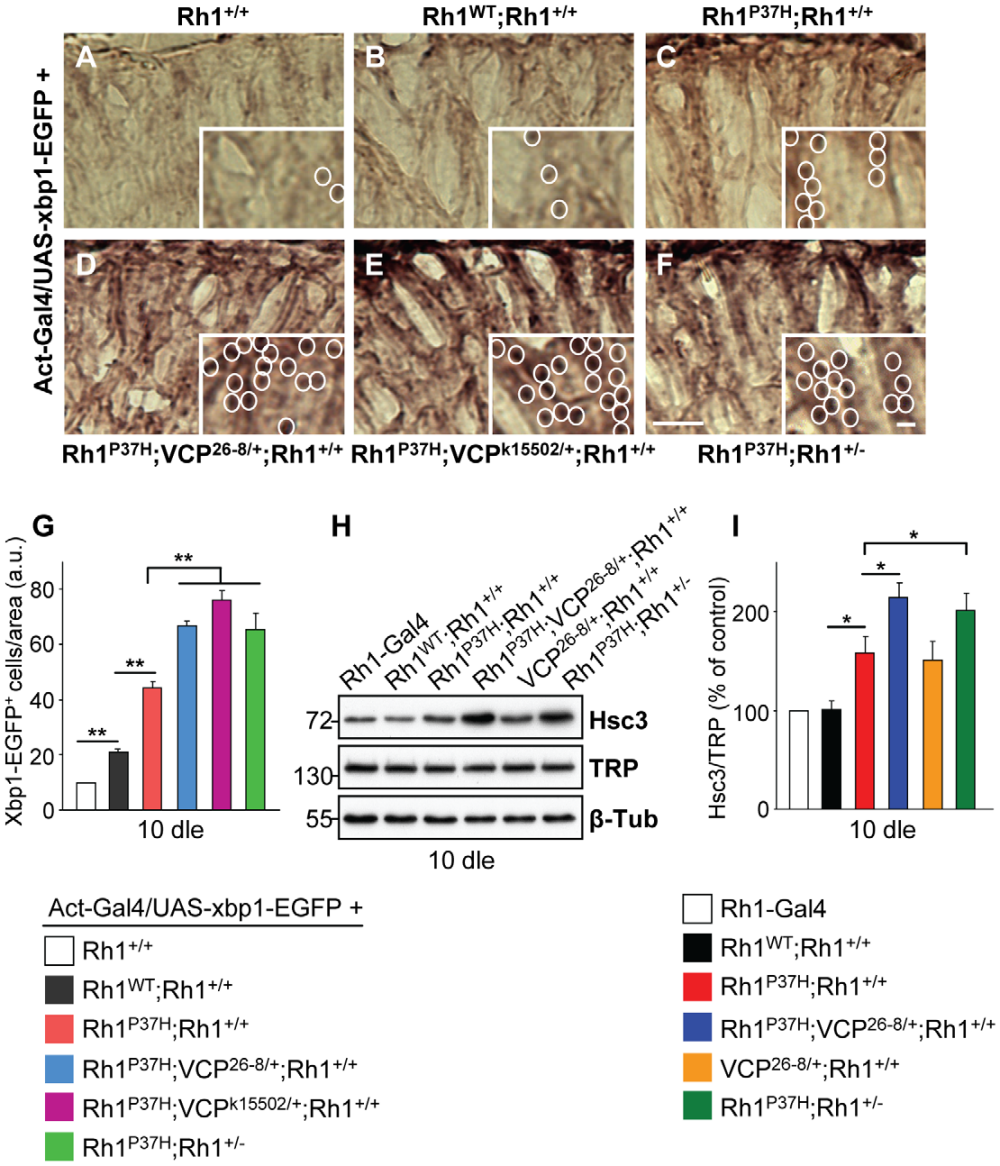
Decreasing *VCP* function increases the activation of the Ire1/Xbp1 UPR pathway in *Rh1^P37H^*-expressing flies. (A–F) Horizontal retinal cryosections from flies after 10 days of light exposure (dle) were stained with an anti-GFP antibody. *xbp1-EGFP* is expressed under the control of *actin* promoter. Unconventional *xbp1* mRNA splicing is increased in the *Rh1^P37H^;Rh1^+/+^* (C) retina while further removal of *VCP* (D,E) or endogenous *Rh1* function (F) strongly enhances *xbp1* unconventional splicing, revealed by EGFP expression. Scale bar is 75 µm and inset is 10 µm. (G) Quantification of Xbp1-EGFP positive nuclei. Results are expressed as number of EGFP-positive nuclei per area from five different flies/group (** p<0.01 t-test). (H) Immunoblot showing the levels of Hsc3 and TRP in flies of indicated genotypes after 10 dle. *Rh1^P37H^;Rh1^+/+^* retinas exhibit more Hsc3 compared to control and *Rh1^WT^;Rh1^+/+^* retinas, while further removal of *VCP* or endogenous *Rh1* function leads to a further increase in Hsc3 levels. β-Tubulin (β-Tub) served as loading control. (I) Quantification of Hsc3 levels (normalized to TRP levels) was averaged from three independent experiments (* p<0.05 t-test).

Accumulation of misfolded proteins in the ER activates the UPR; the unconventional splicing of *xbp1* mRNA is favored during the UPR, generating a transcription factor that activates stress response genes, including the ER stress sensor and chaperone Hsc3/BiP [37], [42], [43]. We tested for the occurrence of *xbp1* mRNA unconventional splicing by inducing ubiquitous expression of an *UAS-xbp1-EGFP* construct, which activates EGFP expression only after unconventional splicing [37]. We stained retinas using a GFP-specific antibody [20], [37] and determined the density of GFP-positive profiles for the different genotypes. We found that Xbp1-EGFP activation is increased in *Rh1^P37H^;Rh1^+/+^* retinas relative to *Rh1^WT^;Rh1^+/+^* and control retinas (Figure 4A–4C and 4G). *Rh1^P37H^;Rh1^+/+^* flies carrying *VCP^26-8^*, *Rh1^−^* or the stronger *VCP* loss-of -function (LOF) allele *VCP^k15502^* [40] displayed an increased density of GFP positive profiles in the retina (Figure 4D–4G; n>5 eyes/genotype), consistent with them having increased levels of misfolded oligomeric Rh1 (see Figure 2E and Figure 3A–3D). We then determined the levels of the Hsc3/BiP chaperone in the retina, as an independent read-out for ER stress [18], [20], [37]. Using a Hsc3-specific antibody [37], we found that *Rh1^P37H^;Rh1^+/+^*retinas display a 50% increase in Hsc3 levels relative to *Rh1^WT^;Rh1^+/+^* retinas, starting from P1 (Figure S6F and S6G). Moreover, as previously seen for Xbp1-EGFP activation, *Rh1^P37H^;Rh1^+/+^* flies carrying *VCP^26-8^*, *Rh1^−^* or *VCP^k15502^* alleles displayed increased Hsc3 levels compared to control flies and to *Rh1^P37H^;Rh1^+/+^* carrying WT alleles of *VCP* or *Rh1* (Figure 4H and 4I and data not shown; results from 3 independent crosses were averaged). Therefore, the increased levels of misfolded Rh1 seen after partial removal of *VCP* or endogenous *Rh1* function were associated with increased activation of the Ire1/Xbp1 UPR pathway.

### Suppression of *Rh1^P37H^*-Induced Retinal Degeneration by *VCP* Loss-of-Function Alleles

**Figure 5:**
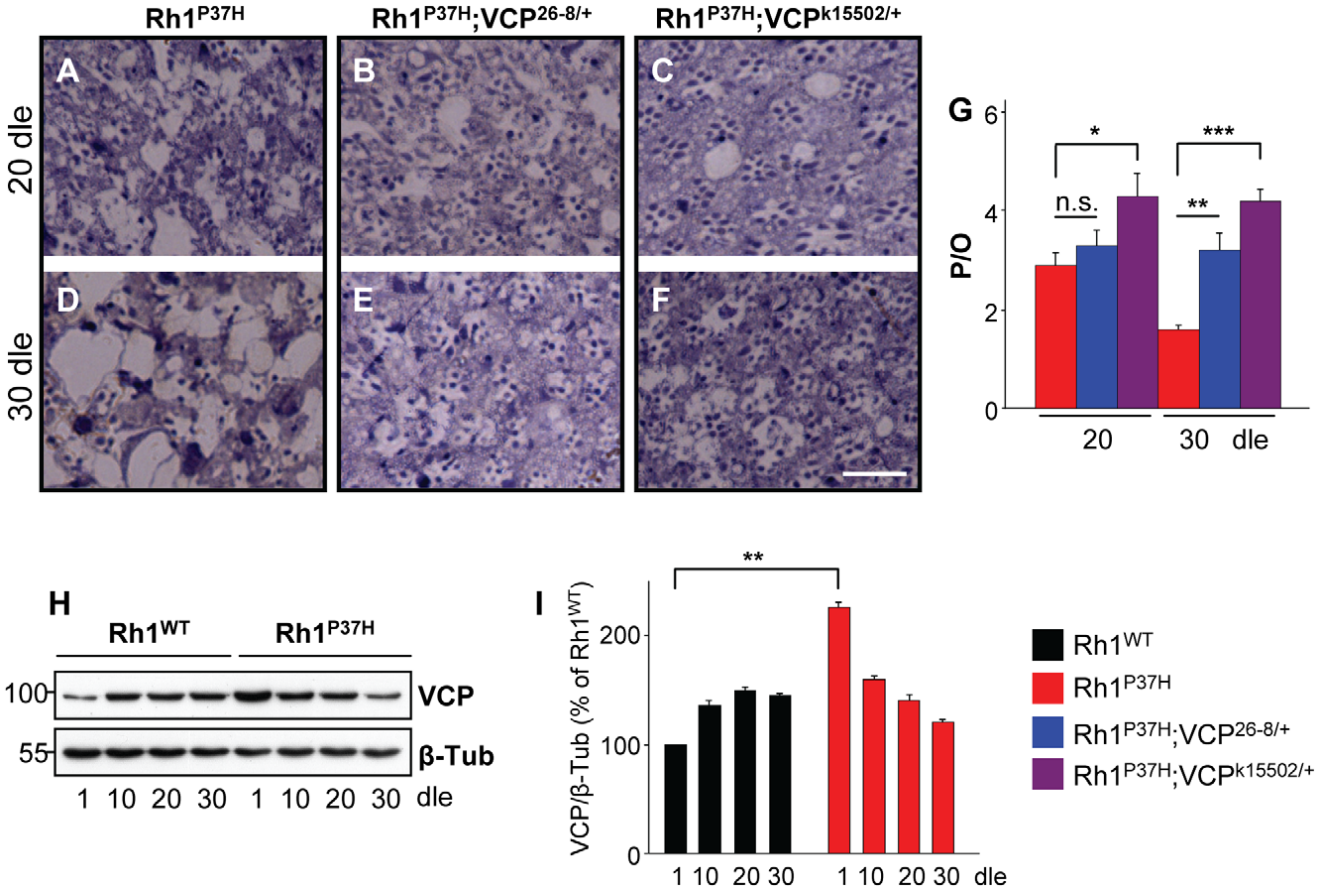
Suppression of *Rh1^P37H^*-induced retinal degeneration by *VCP* loss-of-function alleles. (A–F) Photomicrographs of toluidine blue-stained semithin eye sections of *Rh1^P37H^;Rh1^+/+^* (A,D), *Rh1^P37H^;VCP^26-8/+^;Rh1^+/+^* (B,E) and *Rh1^P37H^;VCP^k15502/+^;Rh1^+/+^* (C,F) flies after 20 (A–C) or 30 (D–F) days of light exposure (dle). Scale bar is 50 µm. (G) Quantification of average number of photoreceptors/ommatidium (P/O) (n>7 animals/group, * p<0.05, ** p<0.01 and *** p<0.001 t-test). Decreasing *VCP* function potently suppresses retinal degeneration caused by Rh1^P37H^. (H) Immunoblot revealing the levels of VCP in *Rh1^WT^;Rh1^+/+^* and *Rh1^P37H^;Rh1^+/+^* retinas, exposed to light for increasing durations. *Rh1^P37H^;Rh1^+/+^* display a 2.25-fold increase of VCP levels relative to *Rh1^WT^;Rh1^+/+^* retinas after 1 dle. β-Tubulin (β-Tub) served as loading control. (I) Quantification of VCP expression levels revealed higher VCP levels in *Rh1^P37H^;Rh1^+/+^* flies after 1 dle. VCP levels were averaged from three independent experiments (** p<0.01 t-test).

We next investigated the effect of decreasing *VCP* function on *Rh1^P37H^*-induced PN degeneration. We found that *Rh1^P37H^;Rh1^+/+^* and *Rh1^P37H^;VCP^26-8/+^;Rh1^+/+^* retinas showed a similar degree of PN degeneration at P20 (Figure 5A, 5B, and 5G), while further degeneration (at P30) in the *Rh1^P37H^;Rh1^+/+^* retina was prevented in the *VCP^26-8^* background (Figure 5D, 5E, and 5G). Remarkably, PN degeneration was strongly suppressed in *Rh1^P37H^;Rh1^+/+^* flies carrying the stronger *VCP* LOF allele *VCP^k15502^* (genotype: *Rh1^P37H^;VCP^k15502/+^;Rh1^+/+^*), both at P20 and P30 (Figure 5A, 5C, 5D, 5F, and 5G). Thus, *VCP* inactivation exerts a protective role on *Rh1^P37H^*-mediated retinal degeneration. We then used a VCP-specific antibody to assess the levels of VCP in *Rh1^P37H^;Rh1^+/+^* versus *Rh1^WT^;Rh1^+/+^* flies. We found a small (2-fold) but consistent increase in VCP levels in *Rh1^P37H^;Rh1^+/+^* flies at P1, which was not maintained at P10 (Figure 5H and 5I; results from 3 independent crosses were averaged). Although an initial increase in VCP levels might induce long-term cellular changes that contribute to PN degeneration, and changes in VCP activation status or localization might be pro-apoptotic, our results suggest that differences in VCP levels play a minor, if any, direct pro-apoptotic role in *Rh1^P37H^*-mediated retinal degeneration.

### Pharmacological Inhibition of the VCP/ERAD/Proteasome Axis Suppresses Retinal Degeneration in *Rh1^P37H^*-Expressing Flies

Our finding that VCP inhibition rescues retinal degeneration in *Rh1^P37H^;Rh1^+/+^* flies suggests that inhibition of the ERAD activity might exert long-term protective effects in *Rh1^P37H^* PNs. The tight coupling between VCP and proteasome activities is essential for substrate delivery and clearance during ERAD [23] and interestingly, proteasome activity is required for proper substrate retrotranslocation [15]. To independently test whether inhibition of the VCP/ERAD/proteasome axis is protective for *Rh1^P37H^*-expressing PNs, we used the VCP/ERAD inhibitor Eeyarestatin I (EerI) and the proteasome inhibitor MG132 that we dissolved in fly food. EerI acts on deubiquitinating enzymes that function downstream of VCP during ERAD and therefore inhibits ERAD-associated VCP functions [44], [45]. MG132 is a classical proteasome inhibitor and potently inhibits the proteasome in *Drosophila* S2 cells [46], [47]. We measured the proteasome activity on head extracts (see Text S1) from control flies to which MG132 was added after lysis and determined that MG132 potently inhibits the activity of the proteasome (Figure S7).

**Figure 6:**
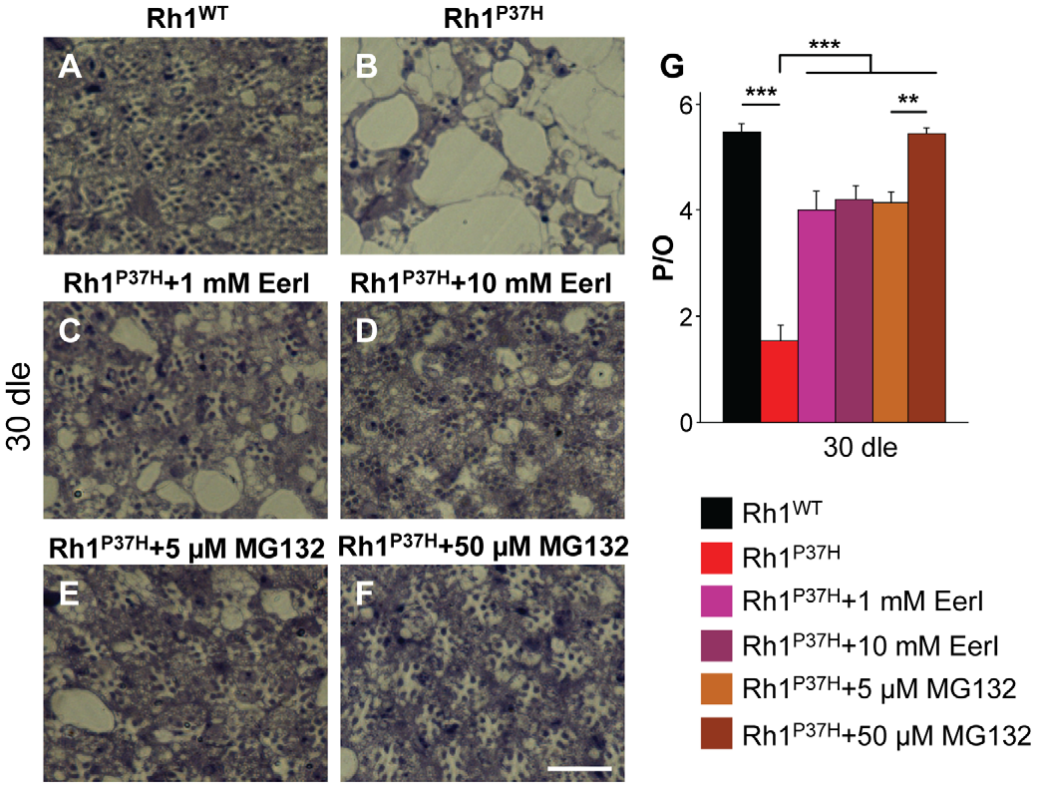
Rescue of *Rh1^P37H^*-induced retinal degeneration after inhibiting the VCP/ERAD (EerI) or the proteasome (MG132) activity. (A–F) Photomicrographs of toluidine blue-stained semithin eye sections of *Rh1^WT^;Rh1^+/+^* flies fed on control food (A); *Rh1^P37H^;Rh1^+/+^* flies fed on control food (B); *Rh1^P37H^;Rh1^+/+^* flies fed on food containing 1 mM (C), or 10 mM (D) EerI; *Rh1^P37H^;Rh1^+/+^* flies fed on food containing 5 µM (E) or 50 µM (F) MG132 after 30 dle. Dle: days of light exposure. Scale bar is 50 µm. (G) Quantification of average number of photoreceptors/ommatidium (P/O) (n>7 animals/group, ** p<0.01 and *** p<0.001 t-test).

Using two doses of EerI (1 mM and 10 mM), we found that partial VCP/ERAD inhibition potently suppressed retinal degeneration in *Rh1^P37H^;Rh1^+/+^* flies after 30 dle (Figure 6B–6D and 6G; n>7 flies/genotype). Both doses displayed the same (partial) suppression of PN degeneration, suggesting that a higher dose is required to achieve a more complete rescue or that EerI only inhibits some, but not all, ERAD-related VCP functions. *Rh1^P37H^;Rh1^+/+^* flies transferred to food containing 5 µM MG132 displayed a partial but robust suppression of PN degeneration after 30 dle (Figure 6B, 6E, and 6G). Remarkably, treatment of *Rh1^P37H^;Rh1^+/+^* flies with 50 µM MG132 led to a very dramatic rescue of retinal degeneration, the average number of photoreceptors/ommatidium reaching 5.5 (Figure 6B, 6F, and 6G; n>7 flies/genotype and >150 ommatidia scored/animal). Taken together, these results indicate that inhibition of the VCP/ERAD/proteasome axis is protective for *Rh1^P37H^*-expressing PNs, further suggesting that excessive retrotranslocation and/or degradation of visual pigment is a critical pro-apoptotic event in the *Rh1^P37H^* retina.

### Partial *VCP* Inactivation Restores Visual Processing in *Rh1^P37H^*-Expressing Flies

**Figure 7:**
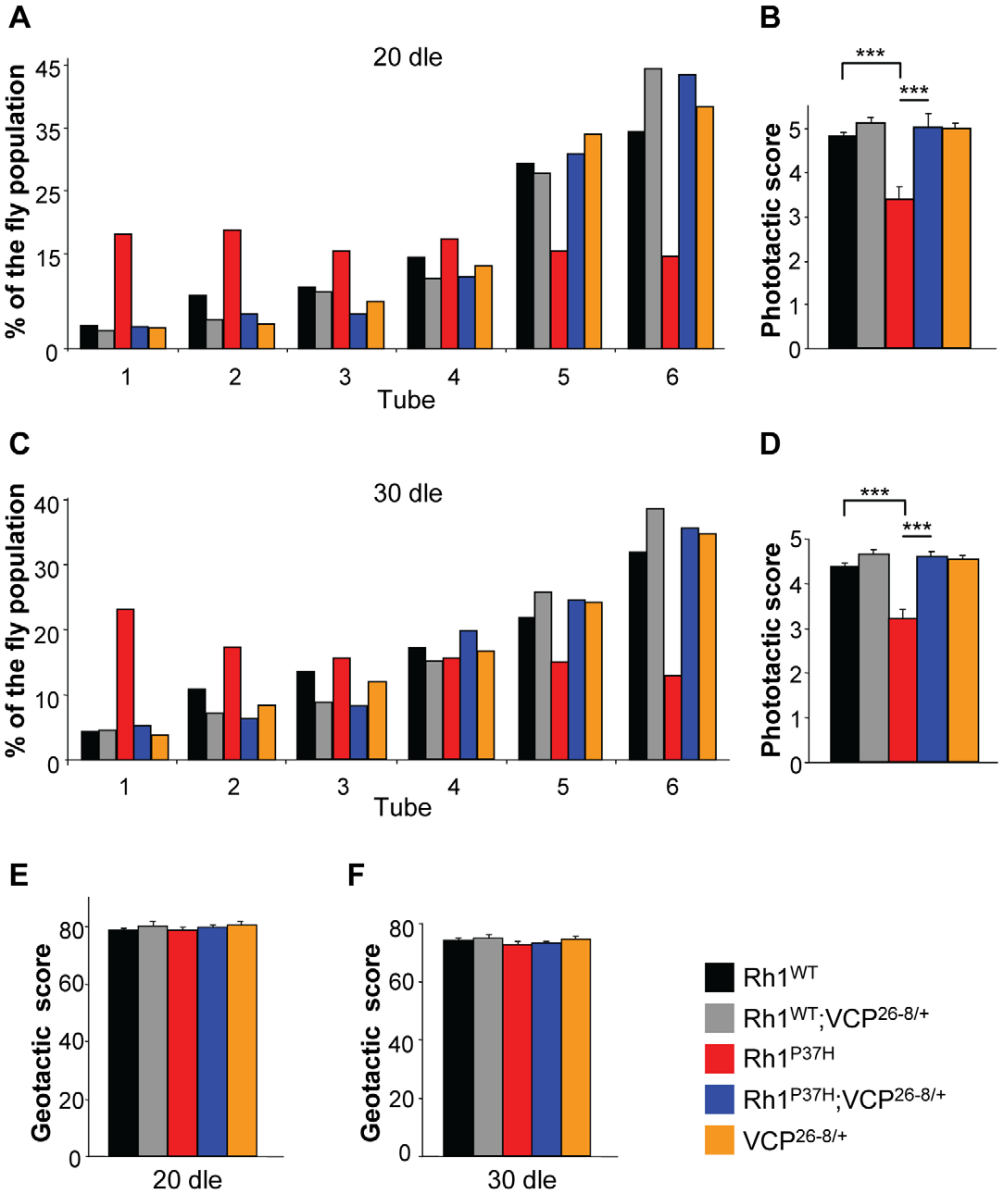
Altered phototaxis in *Rh1^P37H^*-expressing flies is rescued by *VCP* loss-of-function allele. (A,C) Phototaxis histograms after 20 (A) or 30 (C) days of light exposure (dle) revealing the light response of flies of indicated genotypes. (B,D) Phototactic score (PS) after 20 (B) or 30 (D) dle reveals impairment of light response in *Rh1^P37H^;Rh1^+/+^* flies and rescue after decreasing *VCP* function (n = 277–451 flies/genotype, in 3 independent experiments, *** p<0.001 t-test). (E,F) No differences in motor performance in mutant versus control flies chronically exposed to light. Geotactic score of flies of indicated genotypes after 20 (E) or 30 (F) dle. Flies from all genotypes display similar geotactic scores after 20 and 30 dle.

The almost complete restoration of mature Rh1 levels in *Rh1^P37H^;VCP^26-8/+^;Rh1^+/+^* flies prompted us to investigate visual processing in these flies. To assess the capacity of different mutants to process visual information, we performed fast phototaxis measurements [11], [48], [49]. This analysis reflects the average visual functioning of a fly population and scores the percentage of flies that successfully respond to five consecutive light stimulations. Control flies are normally attracted by light, and the analysis of light responses in different groups of mutants serves as an indicator of their retinal integrity. After 20 dle, we found that *Rh1^WT^;Rh1^+/+^*, *Rh1^WT^;VCP^26-8/+^;Rh1^+/+^* and *VCP^26-8/+^;Rh1^+/+^* flies displayed a normal and highly reproducible response to light, i.e. most of these flies moved towards the light source (positive phototaxis) in all five consecutive light stimulations (Figure 7A; n = 277–451 flies/genotype). In contrast, *Rh1^P37H^;Rh1^+/+^* flies were seriously impaired in their light processing capability (Figure 7A) and had a significantly lower phototactic score (PS, defined in Materials and Methods) as compared to *Rh1^WT^;Rh1^+/+^* and control flies (Figure 7B) [11]. Remarkably, in *Rh1^P37H^;VCP^26-8/+^;Rh1^+/+^* flies, positive phototaxis was dramatically improved, consistent with these flies having normal levels of mature Rh1 (Figure 7A and 7B; see Figure S6D). We obtained similar results after 30 dle (Figure 7C and 7D). To rule out that other non-visual related impairments account for the performance of *Rh1^P37H^;Rh1^+/+^* flies in the phototaxis test, we used geotaxis to measure motor functioning in these and all other fly groups, and found that all had a similar motor ability after 20 and 30 dle (Figure 7E and 7F). We found a similar rescue of visual processing in *Rh1^P37H^*-expressing flies when carrying the stronger LOF allele, *VCP^k15502^* (Figure S8H, S8I, S8J); moreover, *Rh1^P37H^*-expressing flies carrying one *Rh1* null allele (*Rh1^−^*) also showed improved phototaxis (Figure S8K, S8L, S8M) although the recovery was partial, reflecting their Rh1 content (Figure 2D). To evaluate whether the protective effect achieved by partial VCP inhibition also extends to other class II *Rh1* mutations, we used another class II mutant, *ninaE^D1^* (*Rh1^S137F^*), which also displays a progressive decline in visual acuity [34]. We found, similarly, that reducing *VCP* activity partially suppressed the loss of PNs in the *ninaE^D1/+^* retina (Figure S8A, S8B, S8C, S8D) and restored the vision deficits displayed by the *ninaE^D1/+^* flies (Figure S8E, S8F, S8G). Thus, reducing *VCP* activity rescues retinal degeneration and blindness induced by a second class II mutant, *ninaE^D1^*.

**Figure 8:**
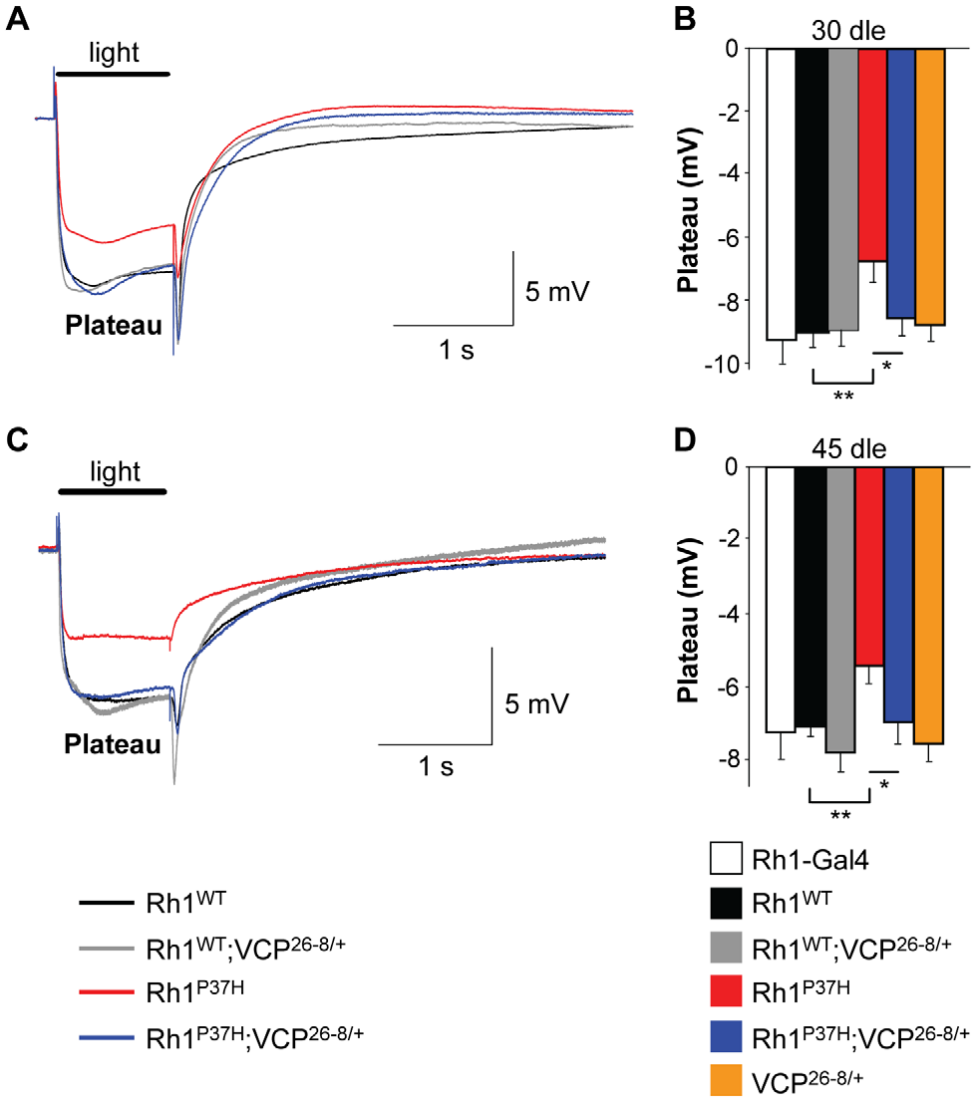
Blindness in *Rh1^P37H^*-expressing flies is rescued in a *VCP* hypomorphic background. (A,C) ERG after 30 (A) or 45 (C) days of light exposure (dle). Plateau represents photoreceptor depolarization. *Rh1^P37H^;Rh1^+/+^* flies (red tracing) display reduced plateau amplitude, while partial *VCP* inactivation restores their light transduction almost to control levels. Time scale: 1 second and plateau amplitude scale: 5 mV. (B,D) Average depolarization amplitudes after 30 (B) or 45 (D) dle. Impaired light transduction in *Rh1^P37H^;Rh1^+/+^* flies is rescued in a *VCP^26-8^* background (n = 12–20 flies/group, * p<0.05 and ** p<0.01 t-test).

Finally, to obtain more direct evidence for a functional rescue of visual processing in *Rh1^P37H^;VCP^26-8/+^;Rh1^+/+^* PNs, we performed electroretinogram (ERG) measurements [11], [39], [50]. We determined that, in contrast to *Rh1-Gal4*, *Rh1^WT^;Rh1^+/+^*, *VCP^26-8/+^;Rh1^+/+^* or *Rh1^WT^;VCP^26-8/+^;Rh1^+/+^* retinas, which displayed a normal photoreceptor depolarization following light stimulation, *Rh1^P37H^*-expressing photoreceptors had a decreased amplitude of photoreceptor depolarization (plateau), both after 30 and 45 dle, consistent with their retinal degeneration phenotype (Figure 8; n = 12–20 flies/genotype). In contrast, light processing in *Rh1^P37H^;VCP^26-8/+^;Rh1^+/+^* photoreceptors was largely rescued, the ERG amplitude values for these flies reaching control levels, both after 30 dle (Figure 8A and 8B) and after 45 dle (Figure 8C and 8D). Taken together, the deleterious effects of mutant *Rh1^P37H^* on light transduction and photoreceptor depolarization are almost completely suppressed by inactivating the function of the ERAD effector *VCP*, indicating that VCP activity promotes neurodegeneration in the *Rh1^P37H^* retina.

## Discussion

In our recent study [16] we found that the ERAD effector VCP interacts with misfolded Rh^P23H^ in mammalian cells and promotes its ATP-dependent retrotranslocation and proteasomal clearance. Here, we have used *Drosophila* transgenics in which Rh1^P37H^ (the equivalent of mammalian Rh^P23H^) induces progressive light- and age-dependent neurodegeneration in PNs [11] and manipulated the levels of the endogenous Rh1, as well as the activity of VCP. We found that misfolded Rh1^P37H^ induces deleterious effects in PNs only in the presence of its WT endogenous counterpart. These deleterious effects include an excessive retrotranslocation and/or degradation of Rh1, mediated by VCP. We suggest that the VCP/ERAD/proteasome axis is a component of *Rh1^P37H^*-induced toxicity in PNs.

### Proteostasis Defects in the *Rh1^P37H^* Retina

As a first step toward a detailed characterization of Rh1^P37H^ proteostasis defects and their relevance to PN integrity, we evaluated the detergent solubility of Rh1 species extracted from transgenic *Rh1^P37H^;Rh1^+/+^* and *Rh1^WT^;Rh1^+/+^* retinas. In detergent-soluble fractions, we observed an early depletion of the misfolded Rh1^P37H^ (already after 1 dle), which suggests maturation defects. These maturation defects were only seen at light (Figure S3C) and were alleviated after partial *VCP* inactivation (Figure 3G), suggesting that light-stimulated visual activity might impact on the maturation of Rh1^P37H^ by promoting VCP-mediated ERAD of Rh1^P37H^.

The detergent-insoluble fractions from transgenic retinas were enriched in Rh1 oligomeric species that migrated at approximately 250–300 kDa - thus presumably containing 6–10 Rh1 molecules. After 20 dle, *Rh1^P37H^* and *Rh1^WT^*-expressing flies displayed high levels of Rh1 oligomers, and oligomer traces were also detected in control (*Rh1-Gal4*) flies; this might be potentially due to reduced folding capacity of the ER during aging (that can be further impaired by protein overexpression or misfolding). Since *VCP* inactivation (which suppressed degeneration) also increased the levels of Rh1 oligomers and both *Rh1^WT^* and *Rh1^P37H^*-expressing flies (at P20-P30) displayed high levels of oligomers (despite clear differences in retinal integrity), the presence of these Rh1 oligomers is probably not the cause of retinal degeneration in the *Rh1^P37H^;Rh1^+/+^* retina. We found a complete absence of mutant Rh1^P37H^ from Rh1-containing oligomers in *Rh1^P37H^* flies, while *VCP* inactivation led to accumulation of Rh1^P37H^-containing oligomers (Figure 3C and 3D and Figure S6C). The fact that we detected Rh1^P37H^ oligomers (hsv positive; Figure 3C and 3D) in *Rh1^P37H^;VCP^26-8/+^;Rh1^+/+^* retina suggests that the absence of Rh1^P37H^ from Rh1 oligomeric species in the *Rh1^P37H^;Rh1^+/+^* retina is due to Rh1^P37H^ degradation, and not to failure to detect the hsv tag. The increased levels of hsv-labeled Rh1^P37H^ oligomers in the insoluble fraction from the *Rh1^P37H^;VCP^26-8/+^;Rh1^+/+^* retina and the activation of the Ire1/Xbp1 ER stress pathway under these conditions (suggesting that these species accumulated in the ER; Figure 4) suggest that VCP activity promotes degradation of Rh1^P37H^, probably by mediating its retrotranslocation from the ER into the cytosol [16], [24], [27]. However, VCP might also promote autophagic degradation of proteins [51]–[53] and it remains to be determined whether Rh1^P37H^ is also degraded by autophagy and whether VCP is involved in this process.

Our experimental protocol for extraction and detection of Rh1-containing insoluble species might not allow us to infer the true nature of Rh1-containing insoluble species *in vivo*. First, the detergent used during extraction might perturb higher-order aggregates (e.g. protofibrils, fibrils) causing them to disassemble during extraction; in addition, different detergents can lead to the extraction of different Rh oligomeric species [54], [55]. Second, Rh1 is able to form oligomers *ex vivo*, during sample preparation. Third, insoluble higher-order aggregates might not enter the SDS-PAGE gel, and remain undetected. Another limitation of our biochemical approach is the lack of information concerning the subcellular localization of Rh1-containing oligomers or aggregates. Further studies using different extraction protocols and/or more powerful biophysical approaches (e.g. spectroscopy using fluorescent probes, fluorescence resonance energy transfer, atomic force microscopy [54], [56]) but also the development of antibodies highly specific to various Rh1 epitopes [57] might help to precisely determine the composition and subcellular localization of Rh1-containing species in the diseased retina.

### Endogenous Rh1 Is Required for *Rh1^P37H^* Toxicity

We found that PN degeneration in the *Rh1^P37H^;Rh1^+/+^* requires an environment in which both mutant Rh1^P37H^ and endogenous Rh1 are present. Thus, the *Rh1^P37H^*-mediated neurodegeneration was strongly suppressed in *Rh1* null background, suggesting that the *Rh1^P37H^* allele is not toxic by itself nor is it sufficient to cause cell death in the absence of its WT counterpart. Transgenic *Rh1^WT^;Rh1^+/+^* flies displayed minor degenerative signs in the retina, suggesting that WT Rh1 alone is not sufficient to cause degeneration. Similar conclusions were also reached with another class II Rh1 mutant, *ninaE^D1^* [34].

Experiments performed in transgenic mice overexpressing a triple (P23H, V20G, P27L) *Rh* mutant (*Rh^GHL^*) indicated that decreased levels of endogenous Rh accelerated the rate of retinal degeneration [58]. These findings are in apparent conflict with our present observations and with those made using *ninaE^D1^* flies [34]. Both *Rh^GHL^* mice and *Rh1^P37H^* flies express the mutant Rh at moderate levels (10–25% and 51% of normal levels, respectively), suggesting that toxicity was not due to overexpression. Several differences between the two models might explain these findings. First, Rh^GHL^ is a triple Rh mutant and might display different properties when compared to Rh^P23H^; the analysis of *Rh^P23H^* transgenic mice [59] will address this possibility. Second, the subcellular localization of the mutant Rh was different, Rh^GHL^ being properly targeted to the membrane (in the *Rh^GHL^;Rh^+/+^* retina), while Rh1^P37H^ showed severe maturation defects (*Rh1^P37H^;Rh1^+/+^* retina). Interestingly, removal of endogenous Rh function impacted (in opposite ways) on the maturation of misfolded Rh, and Rh maturation defects caused a more severe pathology in both cases. It would be thus interesting to evaluate the effect of VCP heterozygosity in the *Rh^GHL^* retina. Third, the most notable difference is that *Rh^GHL^*-mediated degeneration does not require exposure to light (but can be further accelerated by light [8], [60], [61]), while the *Rh1^P37H^*-induced degeneration is strictly light-dependent. Interestingly, we also found that light exposure impairs the maturation of Rh1^P37H^, suggesting a possible interaction between visual processing and the protein quality control machinery in controlling Rh1 homeostasis. Future studies examining *Rh^P23H^* RP patients will establish the role played by light in RP.

### VCP Activity Promotes Retinal Degeneration and Blindness in the *Rh1^P37H^* Retina

Partial *VCP* inactivation rescued retinal degeneration and blindness in *Rh1^P37H^* flies; moreover, the rescuing effect of *VCP* inactivation also extends to another class II Rh1 mutation, *ninaE^D1^* (Figure S8). Two lines of evidence suggest that the effect of VCP on the *Rh1^P37H^* pathology involves its activity as ERAD effector. First, the levels of VCP were found to be increased very early (after 1dle) in *Rh1^P37H^*-expressing flies, well before the onset of retinal degeneration; this suggests that VCP has no direct cell death-promoting activity. Since UPR activation might induce ERAD genes [19], the increase in VCP levels might have been caused by the early activation of the Ire1/Xbp1 pathway. Second, our pharmacological treatment with the EerI inhibitor potently suppressed retinal degeneration in *Rh1^P37H^* flies. EerI was reported to inhibit the activity of deubiquitinating enzymes acting downstream of VCP during ERAD, therefore inhibiting an ERAD-associated VCP activity [44], [45]. Our pharmacological treatment with the proteasome inhibitor MG132 also led to a dramatic suppression of retinal degeneration in the *Rh1^P37H^* retina and the proteasome was shown to be tightly coupled to substrate retrotranslocation during ERAD [15], [62], [63]. The very potent rescuing effect on PN degeneration after genetic inactivation of *VCP*, or after pharmacological treatment with EerI or MG132 suggests that excessive retrotranslocation and/or degradation of visual pigment is pathogenic for *Rh1^P37H^* PNs.

Our results suggest that mechanism underlying *Rh1^P37H^* dominance in the retina is not a DN effect on the maturation of WT Rh1; the endogenous Rh1 matured normally in *Rh1^P37H^* flies (see also [11]) and no decrease in the levels of mature Rh1 preceded PN degeneration in *Rh1^P37H^* flies (Figure 1G). Excessive VCP-mediated degradation of visual pigment might be an underlying mechanism of *Rh1^P37H^* dominance in PNs. The Rh1^P37H^ mutant is not toxic by itself (Figure 2) but requires the endogenous Rh1, VCP activity and light exposure to cause massive PN degeneration. All components of this quadruple interaction network are necessary for toxicity, as elimination of any of them mitigates neurodegeneration. How do these network components relate to each other? The hypothesis we favor is that light-initiated visual processing affects the maturation and stability of the mutant Rh1^P37H^, leading to its enhanced VCP-dependent degradation, with deleterious consequences for the cell. During visual processing, the rhabdomeric WT Rh1 undergoes repeated cycles of internalization [64] and new Rh1 synthesis might re-supply the rhabdomeres. The synthesis and trafficking of WT Rh1 through the ER might be enhanced following light exposure, and this might saturate the ER folding machinery. Excess WT Rh1 might fail to fold, and could self-assemble (to form oligomers, as seen in Figure 1F). At the same time, misfolded Rh1^P37H^ would undergo ERAD at a higher rate, which might be pathogenic. The competition between WT Rh1 and Rh1^P37H^ for the ER folding machinery might thus control PN survival. It would be interesting to determine how light exposure and dark rearing impact on the activity of ERAD, in the presence of WT and/or P37H Rh1.

How do enhanced retrotranslocation and/or degradation of visual pigment lead to PN degeneration in the *Rh1^P37H^* retina? Excessive retrotranslocation might activate pro-apoptotic pathways, under conditions of chronic ERAD activity. Excessive Rh1^P37H^ retrotranslocation might also increase the amount of cytosolic Rh1^P37H^, which might aggregate if left undegraded. Cytosolic Rh1^P37H^-containing aggregates might be more toxic than Rh1^P37H^ mono/oligomers located in the ER and might generate yet unidentified pro-apoptotic signals if the cells cannot adapt to aggregate-induced stress; interestingly, several molecules and mechanisms were suggested to mediate the transition from an adaptive stress response to apoptosis, including the CHOP transcription factor, caspase activation, Ca^2+^ release and mitochondrial signaling [7].

A recent study [65] investigated another *Rh1* allele (*ninaE^G69D^*) which, although not found in RP patients, has been classified as class II Rh mutation [33]. Overexpression (*Rh1* promoter driven) of ERAD members *Hrd1* and *EDEM2* partially rescued late-onset PN degeneration and loss of mature Rh1 in *ninaE^G69D^* flies. Interestingly, reduced *Hrd1* or *EDEM2* function (by RNAi) also rescued mature Rh1 levels, although its effect on PN degeneration was not assessed. These observations might hint at different mechanisms of dominance or different levels of UPR activation among the different Rh1 alleles (see [7]). Another possibility is that early clearance of the mutant Rh1 (via enhanced ERAD) might have long-term protective effects [65]. Therefore, the manipulation of ERAD activity for therapeutic purposes should take into account the temporal profile of Rh aggregation and the relative contribution of endogenous Rh to oligomeric species/aggregates associated with each individual Rh dominant mutation.

Recent evidence suggests that VCP is also a mediator of autophagy, the second major route for protein clearance in cells [51]–[53]. Autophagy and the ubiquitin-proteasome system (UPS) appear to be coordinated processes, with the autophagy being upregulated upon proteasome impairment [66]. The enhanced autophagy that follows UPS inactivation requires the cytoplasmic histone deacetylase HDAC6 [67] and interestingly, HDAC6 interacts with VCP to determine the fate of ubiquitinated misfolded proteins [68]. Inhibition of VCP might decrease autophagic degradation of Rh1 and confer neuroprotection in our *Rh1^P37H^* flies; however, proteasomal inhibition (that should cause enhanced autophagy) was neuroprotective for the *Rh1^P37H^* retina (Figure 6) and we detected no significant impairment of proteasomal function in *Rh1^P37H^* flies (A.Gr. and M.U., unpublished observations). These conflicting scenarios and the recent failure of autophagy induction to suppress *Rh1^RH27^*-mediated retinal pathology in *Drosophila* [69] argue for additional studies to address the relevance of autophagy-promoting VCP activity to retinal degeneration caused by misfolded Rh.

### Promoting Neuroprotection in the *Rh^P23H^* Retina

Inhibition of *VCP* activity or reduced dosage of the endogenous *Rh1* conferred neuroprotection for *Rh1^P37H^*-expressing PNs, and were associated with increased UPR activation, via the Ire1/Xbp1-mediated Hsc3/BiP production (Figure 2, Figure 4, and Figure 5). Since VCP/ERAD/proteasome inactivation might upregulate other pro-survival pathways, it remains to be determined whether Ire1/Xbp1/Hsc3 activation mediates neuroprotection in the *Rh1^P37H^* retina. It is interesting to note that Rh1 accumulation within the ER was recently shown to induce a moderate activation of the Ire1/Xbp1 pathway which induced long-term pro-survival effects, via inhibition of caspase activation and induction of an antioxidant response [20]. Another study found that subretinal delivery of adeno-associated viruses expressing BiP prevented *Rh^P23H^*-mediated retinal degeneration in rats by suppressing the production of the pro-apoptotic protein CHOP [70]. However, it should be noted that, besides activating Ire1/Xbp1, UPR can also promote apoptosis via two independent pathways [18]. Thus, a better understanding of the differential regulation of UPR pathways during ER stress might provide further clues about the regulation of cellular survival versus apoptosis in PNs expressing misfolded Rh^P23H^.

Is partial *VCP* inactivation able to protect against retinal degeneration in *Rh^P23H^*-linked RP? We found that *VCP* inactivation prevents neurodegeneration in *Rh1^P37H^* flies and VCP inhibition is also sufficient to rescue mutant CFTR from degradation and to partially restore CFTR function in a cellular model of cystic fibrosis [28]. However, mutations that impair VCP function are associated with inclusion body myopathy associated with Paget disease of bone and frontotemporal dementia (IBMPFD) [71], [72]. It remains therefore to be determined whether changes in VCP function suppress some pathologies and enhance others. One interesting experiment is the evaluation of the effect of VCP and proteasome inactivation in the *Rh^P23H^* mouse retina, preferably using local delivery. An improved understanding of the molecular machinery linking Rh quality control and the networks regulating PN maintenance might uncover cellular targets acting specifically in the retina, thus facilitating the therapeutic approaches for RP.

## Materials and Methods

### *Drosophila* Stocks

*Drosophila* lines *p(w^+^ Rh1-Rh1^WT^)*, *p(w^+^ Rh1-Rh1^P37H^)*, *p(w^+^ Rh1-Rh1^P37H-hsv^)* and *p(w+ Rh1-Gal4)* lines referred to as *Rh1^WT^;Rh1^+/+^*, *Rh1^P37H^;Rh1^+/+^*, *Rh1^P37H-hsv^;Rh1^+/+^* and *Rh1-Gal4* were previously described [11]. *ninaE^D1^ (ninaE^S137F^)* and *p(ry^+^ rh1-Rh1^WT-hsv^)* referred to as *Rh1^WT-hsv^;Rh1^+/+^* were a gift kind from J.E. O'Tousa. *ter94^26-8^* hypomorphic allele, referred to as *VCP^26-8^*, was kindly provided by E. Goldstein. *UAS-xbp1-EGFP* was a generous gift from H.D. Ryoo. *Actin-Gal4*, *ninaE*-*null I17* (referred to as *Rh1^−^*), *ter94^k15502^* (referred to as *VCP^k15502^*, strong LOF allele) and *w^1118^* flies were from the Bloomington stock center. Flies were raised on standard cornmeal agar medium, under moderate continuous illumination at 25°C. Moderate illumination was obtained by using photosynthetic fluorescent tubes (in total 170 cd/m^2^). Fly progeny having same eye pigmentation was used during the study.

### Western Blotting

WB was performed as described [15], [73], [74]. Briefly, 30 fly heads were homogenized in 60 µl of RIPA modified buffer (20 mM Tris-HCl pH 8.0, 150 mM NaCl, 1 mM EDTA, 1% Triton X-100, 0.1% SDS and 0.5% sodium deoxycholate) supplemented with protease inhibitors (Roche) and phosphatase inhibitors (Sigma-Aldrich). Lysates were centrifuged at 16.000 *g* for 15 minutes at 4°C. For detergent-insoluble factions, pellets were solubilised in 50 µl solution containing 10 mM Tris-HCl pH 7.5, 1% SDS and protease inhibitor cocktail for 8 minutes at room temperature. 100 µl of RIPA modified buffer containing protease inhibitors was added, and the pellets were then sonicated six times (10 seconds each) at 4°C. Following sonication, the fractions were incubated 30 minutes at 4°C. Detergent-insoluble fractions were then centrifuged at 100 *g* for 10 minutes at 4°C and tissue debris were discarded. For Western Blotting, fractions were normalized for total protein using the *Dc* protein assay (Bio-Rad). An equal volume of 2× Laemmli sample buffer was added to fractions separated by 10% SDS-PAGE for detergent-soluble and by 8% SDS-PAGE for detergent-insoluble fractions and electroblotted onto PVDF membranes (GE Healthcare). Immunodetection was performed according to standard techniques using the following primary antibodies: anti-Rh1 (rabbit polyclonal, 1/5000, gift from D.F. Ready) for detecting Rh1 in detergent-insoluble fractions, 4C5 (mouse monoclonal, 1/5000, DSHB) that detects Rh1 in detergent-soluble fractions, anti-ter94/VCP (rat, 1/5000, gift from D. McKearin), anti-hsv (rabbit polyclonal, 1/8000, Sigma), anti-Hsc3 (guinea pig, 1/2000, gift from H.D. Ryoo), anti-Ubiquitin (mouse monoclonal, 1/300, Invitrogen), anti-TRP (1/10000, gift from A. Huber) and anti-β-Tubulin (mouse monoclonal, 1/4000, Chemicon). Secondary antibodies were horseradish peroxidase-coupled (1/8000, Jackson Immunoresearch). Quantification of band intensity after ECL detection was performed using Image Quant TL software.

### Histology and Immunohistochemistry

Heads were dissected and fixed for 24 h in 2.5% glutaraldehyde, phosphate buffered saline (PBS, pH 7.4) and post-fixed in 1% osmium tetroxide in PBS. After a series of ethanol and propylene oxide dehydration, heads were embedded in epoxy resin (Sigma-Aldrich). For toluidine blue staining, semithin (2 µm) sections were stained with 1% toluidine blue [75]. To determine the average number of photoreceptors/ommatidium (P/O), at least l50 ommatidia were scored per animal from at least 4 animals per genotype.

Immunohistochemistry was performed as described [11], [37], heads were dissected and fixed for 15 minutes in 4% formaldehyde, incubated in 10% sucrose during 2 hours and then in 25% sucrose overnight at 4°C, then embedded in cryomedium. 16 µm-thick cryostat sections were fixed for 15 minutes in 4% formaldehyde, permeated with 0.3% Triton-X 100 in PBS (PBST) during 30 minutes and blocked during 1h with 5% normal goat serum in PBST. To detect non-conventional *xbp1* mRNA splicing of the *xbp1-EGFP* construct, we used an anti-GFP antibody (rabbit polyclonal, 1/400, Molecular Probes) diluted in blocking solution. After overnight incubation with primary antibody, an alkaline phosphatase-linked anti-rabbit secondary antibody (1/200, Sigma-Aldrich) was added for 2 hours at room temperature. Sections were washed with alkaline phosphatase buffer (100 mM NaCl, 50 mM MgCl_2_, 100 mM Tris-HCl pH 9.5, 0.1% Tween 20). Then, sections were incubated with BCIP/NBT substrate (Sigma-Aldrich) during 30 minutes, washed in water, air-dried and mounted in a non-aqueous mounting medium (Clarion, Sigma-Aldrich).

### Pharmacological Treatments

Flies were treated with EerI (ChemBridge Corporation, catalog n° 5138427) or MG132 (Sigma-Aldrich) inhibitors dissolved in their food. We used 2 doses of EerI (1 mM and 10 mM) and 2 doses of MG132 (5 µM and 50 µM). These compounds were first dissolved in DMSO and the solution was then added to cooled cornmeal agar fly food. The food was then dispensed into empty vials and allowed to solidify. It was kept at 4°C in the dark for maximum 3 days. The above-mentioned concentrations correspond to the final concentrations in fly food. Flies were transferred in vials containing this modified food right after birth, were reared as described above and transferred to fresh vials every day. The control food contained all the ingredients (including DMSO) except the active compound. After 30 days of light exposure, flies fed on control food (2% DMSO) and on drug food (EerI or MG132 in 2% DMSO) were sacrificed and their retinal integrity was assessed histologically.

### Behavioral Assays

Fast phototaxis was performed as previously described [11]. Briefly, 20 flies were placed in tube 1 of a countercurrent apparatus with six tubes and gently tapped to the bottom of the tube. The apparatus was placed horizontally, the light source was switched on and the flies were allowed to walk toward the light during 30 seconds. The flies that moved toward the light were shifted to the second tube and this procedure was repeated five times. At the end of the test, the number of flies was counted in each of the six tubes. Between 277–451 flies were scored per genotype, in 3 independent experiments. The phototactic score (PS) quantifies the visual activity following a weighted equation (*ΣiN_i_)/ΣN_i_*, where *N* is the number of flies in the i^th^ tube.

Geotaxis was used to assess motor performance in flies chronically exposed to light. About 20 flies/tube were gently tapped to the bottom of the tube and then allowed to climb during 20 seconds. We then scored the number of flies that climbed 4 cm or above. Each tube was tested twice. The results represent the% of flies that reached 4 cm or above after 20 seconds.

### Electroretinogram

Electroretinogram analysis was performed as previously described [11]. A tungsten electrode was introduced in the back of the fly head and a glass electrode filled with 3M KCl was introduced through the cornea. Flashes were delivered using a white LED (Nichia, NSPW510BS, emitting from 425 to 750 nm with two peaks at 460 and 560 nm) with a 45° viewing angle, placed at 1.5 cm from the fly eye and controlled via an Analog Out port of a Digidata 1322A (Molecular Devices, USA). After 5 minutes of dark-adaptation, six 1 second light pulses were used to stimulate the eye and their responses averaged. The light intensity reaching the eye (∼300 µW/cm^2^) was chosen as the minimal intensity that consistently produced maximal ERG plateaus.

## Supporting Information

Figure S1 *Rh1^P37H^*-expressing flies show no retinal degeneration when reared in the dark or after a short exposure to light. (A–D) Photomicrographs of toluidine blue-stained semithin adult eye sections of *Rh1^WT^;Rh1^+/+^*(A,C) and *Rh1^P37H^;Rh1^+/+^* flies (B,D) reared in the light until postnatal day 1 (P1) (A,B) or reared in the dark until P30 (C,D). No loss of photoreceptor neurons is observed under these conditions. Scale bar is 50 µm.
(2.17 MB TIF)

Figure S2 Rh1 levels in *Rh1^WT^*-expressing and *Rh1-Gal4* flies exposed to light. (A,B) Immunoblots revealing the abundance of total (endogenous and ectopic) Rh1 in detergent-soluble (A) or insoluble (B) fractions obtained from retina lysates of *Rh1^WT^;Rh1^+/+^* or *Rh1-Gal4* flies exposed to light for the indicated durations. A short exposure of the Rh1 WB is shown (A) in order to better visualize the differences in Rh1 levels between *Rh1^WT^;Rh1^+/+^* and *Rh1-Gal4* flies. β-Tubulin (β-Tub) served as loading control.
(0.68 MB TIF)

Figure S3 *Rh1^WT^* and *Rh1^P37H^* transgene expression and light-induced Rh1^P37H^ maturation defects. (A–C) Immunoblots revealing the abundance of total Rh1 (endogenous and ectopic, Rh1 antibody) (A,B) and ectopic Rh1 (hsv antibody) (C) in detergent-soluble fractions obtained from retinas of *Rh1-Gal4*, *Rh1^WT^;Rh1^+/+^* or *Rh1^P37H^;Rh1^+/+^* flies reared at light (A–C) or in the dark (C) for one day. Misfolded Rh1^P37H^ shows maturation defects in the presence of light (C). (D) Immunoblot showing the levels of total Rh1 (endogenous and ectopic, Rh1 antibody) in detergent-soluble fractions obtained from retinas of flies of indicated genotypes reared at light for 10 days. Complete absence of endogenous Rh1 leads to high expression levels of the mutant *Rh1^P37H^* transgene (see lanes 3 and 4 in Rh1 WB), and under these conditions, the levels of mature Rh1^P37H^ are significantly higher. Please note that (unlike in C, lanes 2 and 6) only ectopic Rh1^P37H^ is present in D (lanes 3 and 4) and that the Rh1 signal therefore indicates the amount of mature Rh1^P37H^ in these retinas. β-Tubulin (β-Tub) served as loading control.
(1.18 MB TIF)

Figure S4 Loss of Rh1^P37H^ in *Rh1^P37H^*-expressing flies exposed to light. (A,B) Immunoblots revealing the abundance of total (endogenous and ectopic, Rh1 antibody) and ectopic (hsv antibody) Rh1 in detergent-soluble (A) or insoluble (B) fractions obtained from retinas of *Rh1^WT^;Rh1^+/+^* or *Rh1^P37H^;Rh1^+/+^* flies exposed to light for the indicated durations. Aggregates were independently labeled with an Ubiquitin-specific antibody (B).
(1.11 MB TIF)

Figure S5 Rescue of retinal degeneration in *Rh1^P37H^*-expressing flies lacking endogenous Rh1. Immunoblot showing the levels of total Rh1 (Rh1 antibody) in detergent-soluble fractions obtained from retinas of flies of indicated genotypes reared at light for 25 days. Rh1^P37H^ transgene expression in a *Rh1* null background (lanes 3 and 4) suppresses retinal degeneration and prevents the loss of Rh1 seen in *Rh1^P37H^;Rh1^+/+^* flies (lane 2). β-Tubulin (β-Tub) served as loading control.
(0.27 MB TIF)

Figure S6 *VCP* inactivation increases Rh1 aggregate load and restores the levels of mature Rh1 in *Rh1^P37H^*-expressing flies. (A) Flies carrying the VCP hypomorphic allele *VCP^26-8^* have reduced levels of VCP. Adult WT and *VCP^26-8/+^* (genotype: *VCP^26-8/+^;Rh1^+/+^*) flies were tested for VCP expression levels using a VCP-specific antibody. VCP levels are significantly reduced in *VCP^26-8/+^* flies, as compared to WT flies. (B,C) Immunoblots showing levels of total (endogenous and ectopic, Rh1 antibody; B) and ectopic (hsv antibody; C) Rh1 aggregates in flies of indicated genotypes after 20 days of light exposure (dle). An Ubiquitin-specific antibody was used to independently label aggregates. The amount of aggregated (endogenous and ectopic) Rh1 increases in *Rh1^P37H^;VCP^26-8/+^;Rh1^+/+^* flies compared to control and mutant *Rh1^P37H^;Rh1^+/+^* flies (B). Aggregates containing the ectopic Rh1^P37H^ are also rescued from degradation after partial *VCP* inactivation (C). (D,E) Immunoblots revealing the levels of total (endogenous and ectopic, Rh1 antibody; D) and ectopic (hsv antibody; E) mature Rh1 in flies of indicated genotypes after 20 dle. There is loss of mature Rh1 in *Rh1^P37H^;Rh1^+/+^* flies, which is rescued when reducing *VCP* function (D). Loss of ectopic mature Rh1^P37H^ in *Rh1^P37H^;Rh1^+/+^* flies is also rescued in *Rh1^P37H^;VCP^26-8/+^;Rh1^+/+^* flies (E). Ectopic Rh1 was hsv-tagged in *Rh1^WT^;Rh1^+/+^* or *Rh1^P37H^;Rh1^+/+^* flies; no hsv signal was detected in *Rh1-Gal4* flies which lacked hsv-tagged Rh1 (E). Please note that 10-fold less protein was loaded for *Rh1^WT^;Rh1^+/+^* flies (E). (F) Immunoblot revealing the levels of Hsc3 in *Rh1^WT^;Rh1^+/+^* and *Rh1^P37H^;Rh1^+/+^* retinas, exposed to light for increasing durations. A 1.5-fold increase of Hsc3 levels is seen in *Rh1^P37H^;Rh1^+/+^* versus *Rh1^WT^;Rh1^+/+^* retinas starting at day 1. β-Tubulin (β-Tub) served as loading control. (G) Quantification of Hsc3 expression levels. The results are expressed as mean percentage compared to Hsc3 levels in *Rh1^WT^;Rh1^+/+^* retinas at day 1 (100%) and were averaged from three independent experiments (** p<0.01 t-test).
(0.70 MB TIF)

Figure S7 The proteasome inhibitor MG132 potently suppresses proteasome activity in *Drosophila*. Head fly lysates were assayed for the proteasome activity in the absence (control, 0.5% DMSO) or presence of the proteasome inhibitor MG132 (50 µM MG132 in 0.5% DMSO). The results are shown as mean percentage compared to proteasome activity levels in fly lysates treated with DMSO (set as 100%) and were averaged from three independent experiments (*** p<0.001 t-test).
(0.05 MB TIF)

Figure S8 Retinal pathology in *ninaE^D1^* and *Rh1^P37H^* flies is rescued by *VCP* or *Rh1* inactivation. (A–C) Photomicrographs of toluidine blue-stained semithin eye sections of WT (A), *ninaE^D1/+^* (B), *VCP^26-8/+^;ninaE^D1/+^* (C) flies after 30 days of light exposure (dle). Scale bar is 50 µm. (D) Quantification of average number of photoreceptors/ommatidium (P/O) (n>6 animals/group, ** p<0.01 and *** p<0.001 t-test). Decreasing *VCP* function suppresses retinal degeneration caused by ninaE^D1^. (E) Phototaxis histogram after 12 dle revealing the light response of flies of indicated genotypes. *ninaE^D1/+^* flies show visual impairment relative to WT flies, while *VCP^26-8/+^;ninaE^D1/+^* flies display rescue of visual acuity. Between 250–300 flies were scored/genotype. (F) Phototactic score (PS) of WT, *ninaE^D1/+^* and *VCP^26-8/+^;ninaE^D1/+^* flies after 12 dle (n = 250–300 flies/group, ** p<0.01 and *** p<0.001 t-test). (G) Geotactic score of WT, *ninaE^D1/+^* and *VCP^26-8/+^;ninaE^D1/+^* flies after 12 dle. Flies from all genotypes display similar geotactic scores. (H) Phototaxis histogram after 20 dle revealing the light response of flies of indicated genotypes. While *Rh1^P37H^;Rh1^+/+^* flies show visual impairment, *Rh1^P37H^;VCP^k15502/+^;Rh1^+/+^* flies have an improved visual acuity. Between 150–200 flies were scored/genotype. (I) PS of *Rh1^P37H^;Rh1^+/+^* and *Rh1^P37H^;VCP^k15502/+^;Rh1^+/+^* flies after 20 dle (n = 150–200 flies/group, *** p<0.001 t-test). (J) Geotactic score of *Rh1^P37H^;Rh1^+/+^* and *Rh1^P37H^;VCP^k15502/+^;Rh1^+/+^* flies after 20 dle. Flies from both genotypes show similar geotactic scores. (K) Phototaxis histogram after 20 dle revealing the light response of flies of indicated genotypes. *Rh1^P37H^;Rh1^+/+^* flies display visual impairment compared to *Rh1^WT^;Rh1^+/+^* flies, while *Rh1^P37H^;Rh1^+/−^* flies have an improved visual acuity. Between 200–230 flies were scored/genotype. (L) PS of *Rh1^WT^;Rh1^+/+^*, *Rh1^P37H^;Rh1^+/+^* and *Rh1^P37H^;Rh1^+/−^* flies after 20 dle (n = 200–230 flies/group, * p<0.05; ** p<0.01 t-test). (M) Geotactic score of *Rh1^WT^;Rh1^+/+^*; *Rh1^P37H^;Rh1^+/+^* and *Rh1^P37H^;Rh1^+/−^* flies after 20 dle. Flies from all genotypes show similar geotactic scores.
(1.16 MB TIF)

Text S1 Supplementary Materials and Methods. Measurement of proteasome activity from fly head extracts.
(0.03 MB DOC)
