# Supplementary figures and images for: Inactivation of VCP/ter94 Suppresses Retinal Pathology Caused by Misfolded Rhodopsin in Drosophila
Source: PLoS Genet. 2010 Aug 26;6(8):e1001075. doi: 10.1371/journal.pgen.1001075 (PMC2928793; doi:10.1371/journal.pgen.1001075)

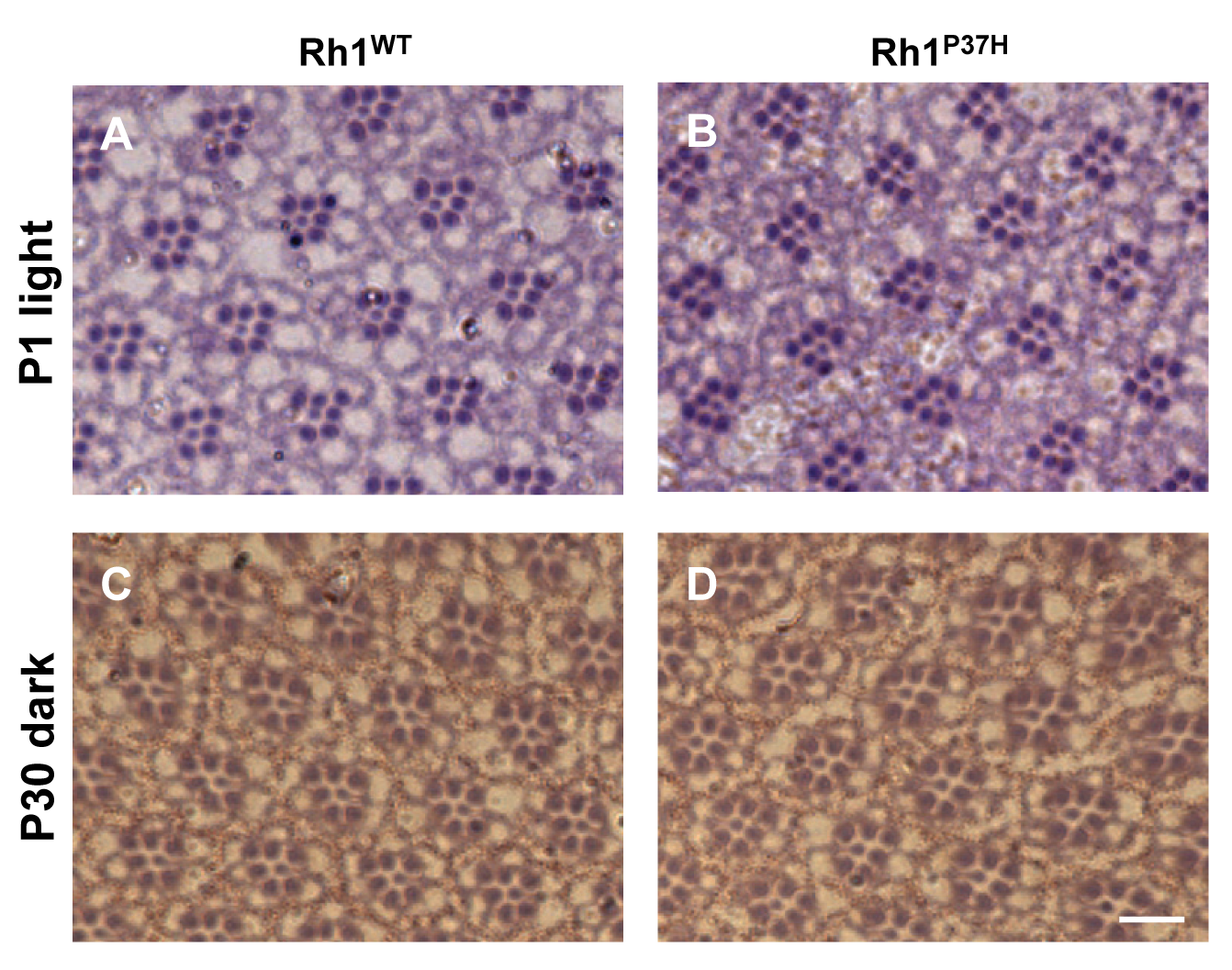

Supplement: Figure S1 — Rh1P37H-expressing flies show no retinal degeneration when reared in the dark or after a short exposure to light. (A–D) Photomicrographs of toluidine blue-stained semithin adult eye sections of Rh1WT;Rh1+/+(A,C) and Rh1P37H;Rh1+/+ flies (B,D) reared in the light until postnatal day 1 (P1) (A,B) or reared in the dark until P30 (C,D). No loss of photoreceptor neurons is observed under these conditions. Scale bar is 50 µm. (2.17 MB TIF) [file pgen.1001075.s001.tif]

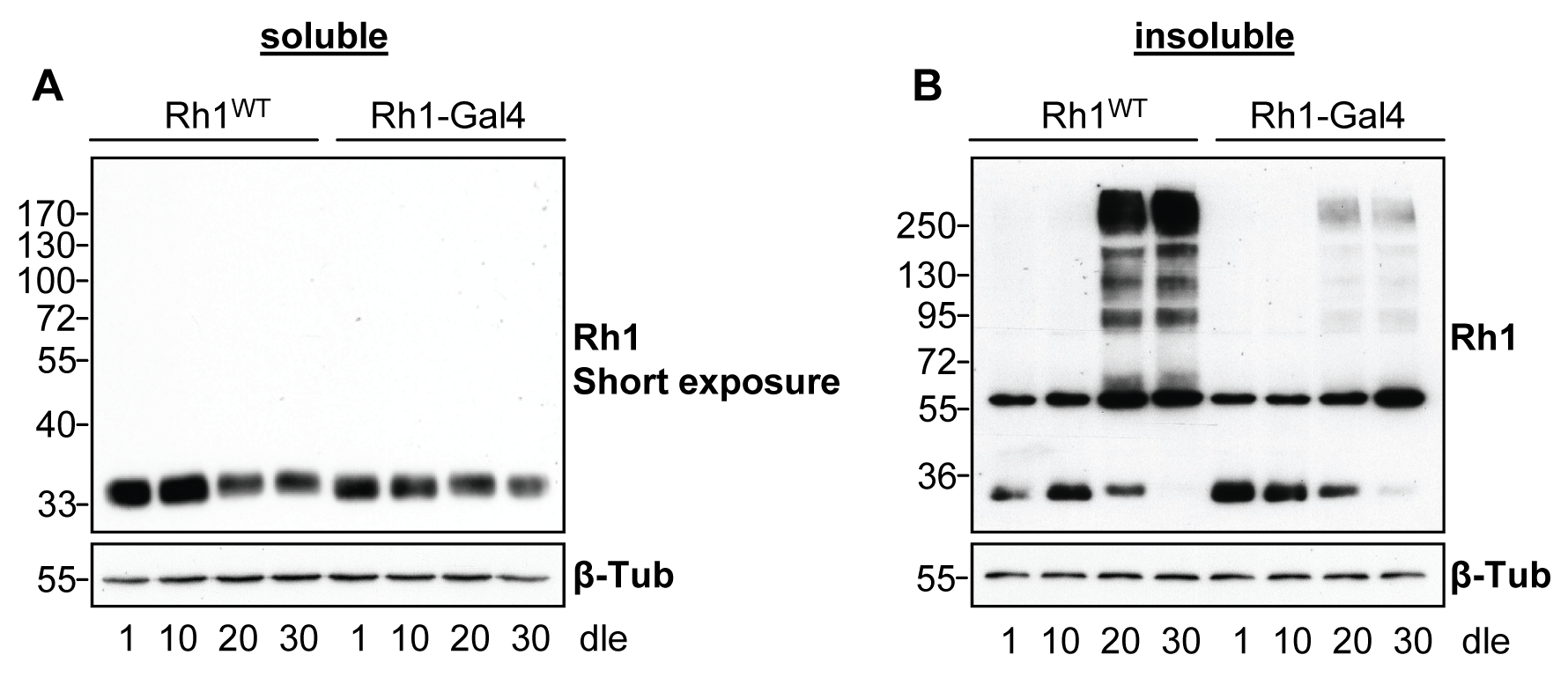

Supplement: Figure S2 — Rh1 levels in Rh1WT-expressing and Rh1-Gal4 flies exposed to light. (A,B) Immunoblots revealing the abundance of total (endogenous and ectopic) Rh1 in detergent-soluble (A) or insoluble (B) fractions obtained from retina lysates of Rh1WT;Rh1+/+ or Rh1-Gal4 flies exposed to light for the indicated durations. A short exposure of the Rh1 WB is shown (A) in order to better visualize the differences in Rh1 levels between Rh1WT;Rh1+/+ and Rh1-Gal4 flies. β-Tubulin (β-Tub) served as loading control. (0.68 MB TIF) [file pgen.1001075.s002.tif]

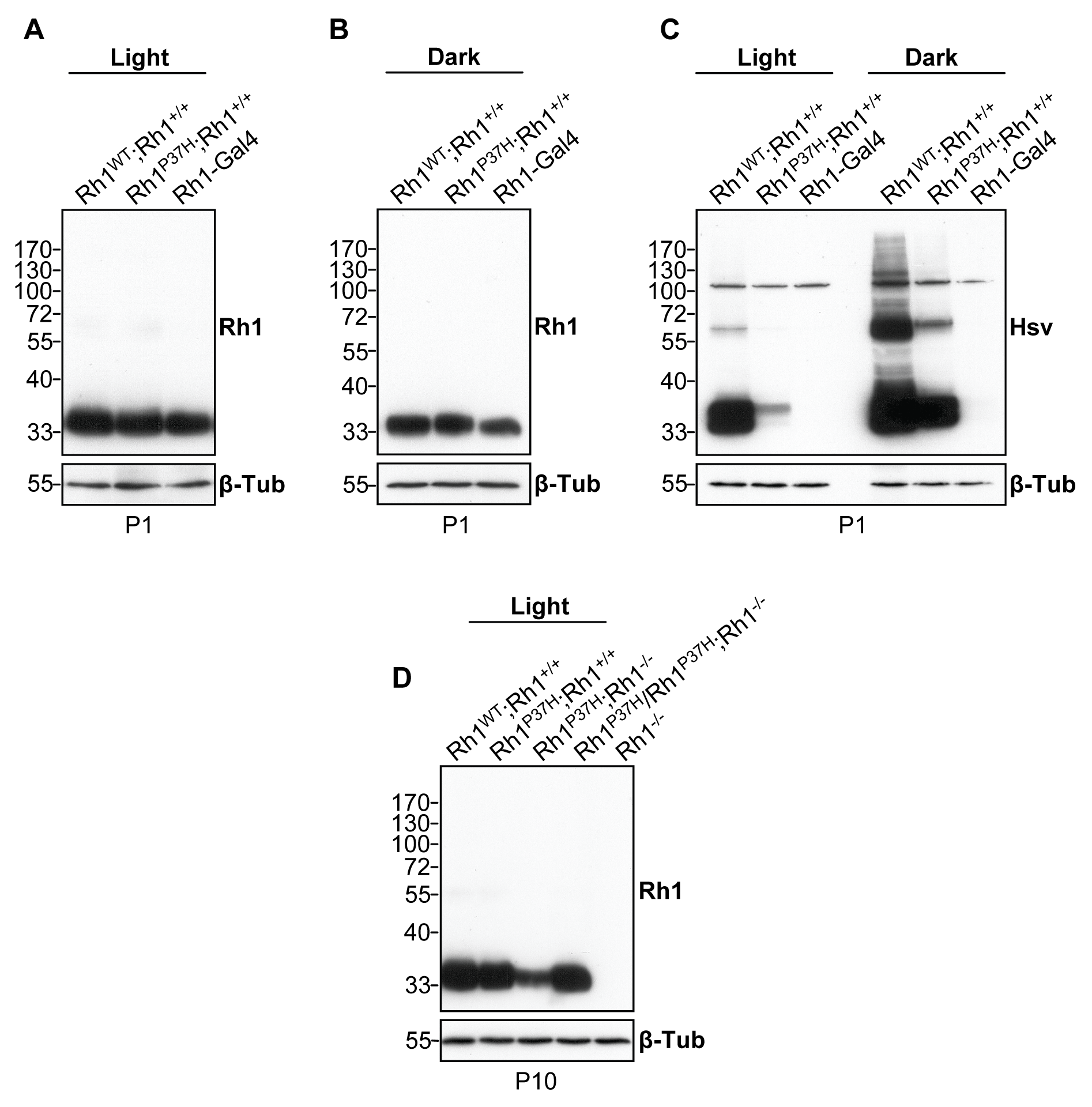

Supplement: Figure S3 — Rh1WT and Rh1P37H transgene expression and light-induced Rh1P37H maturation defects. (A–C) Immunoblots revealing the abundance of total Rh1 (endogenous and ectopic, Rh1 antibody) (A,B) and ectopic Rh1 (hsv antibody) (C) in detergent-soluble fractions obtained from retinas of Rh1-Gal4, Rh1WT;Rh1+/+ or Rh1P37H;Rh1+/+ flies reared at light (A–C) or in the dark (C) for one day. Misfolded Rh1P37H shows maturation defects in the presence of light (C). (D) Immunoblot showing the levels of total Rh1 (endogenous and ectopic, Rh1 antibody) in detergent-soluble fractions obtained from retinas of flies of indicated genotypes reared at light for 10 days. Complete absence of endogenous Rh1 leads to high expression levels of the mutant Rh1P37H transgene (see lanes 3 and 4 in Rh1 WB), and under these conditions, the levels of mature Rh1P37H are significantly higher. Please note that (unlike in C, lanes 2 and 6) only ectopic Rh1P37H is present in D (lanes 3 and 4) and that the Rh1 signal therefore indicates the amount of mature Rh1P37H in these retinas. β-Tubulin (β-Tub) served as loading control. (1.18 MB TIF) [file pgen.1001075.s003.tif]

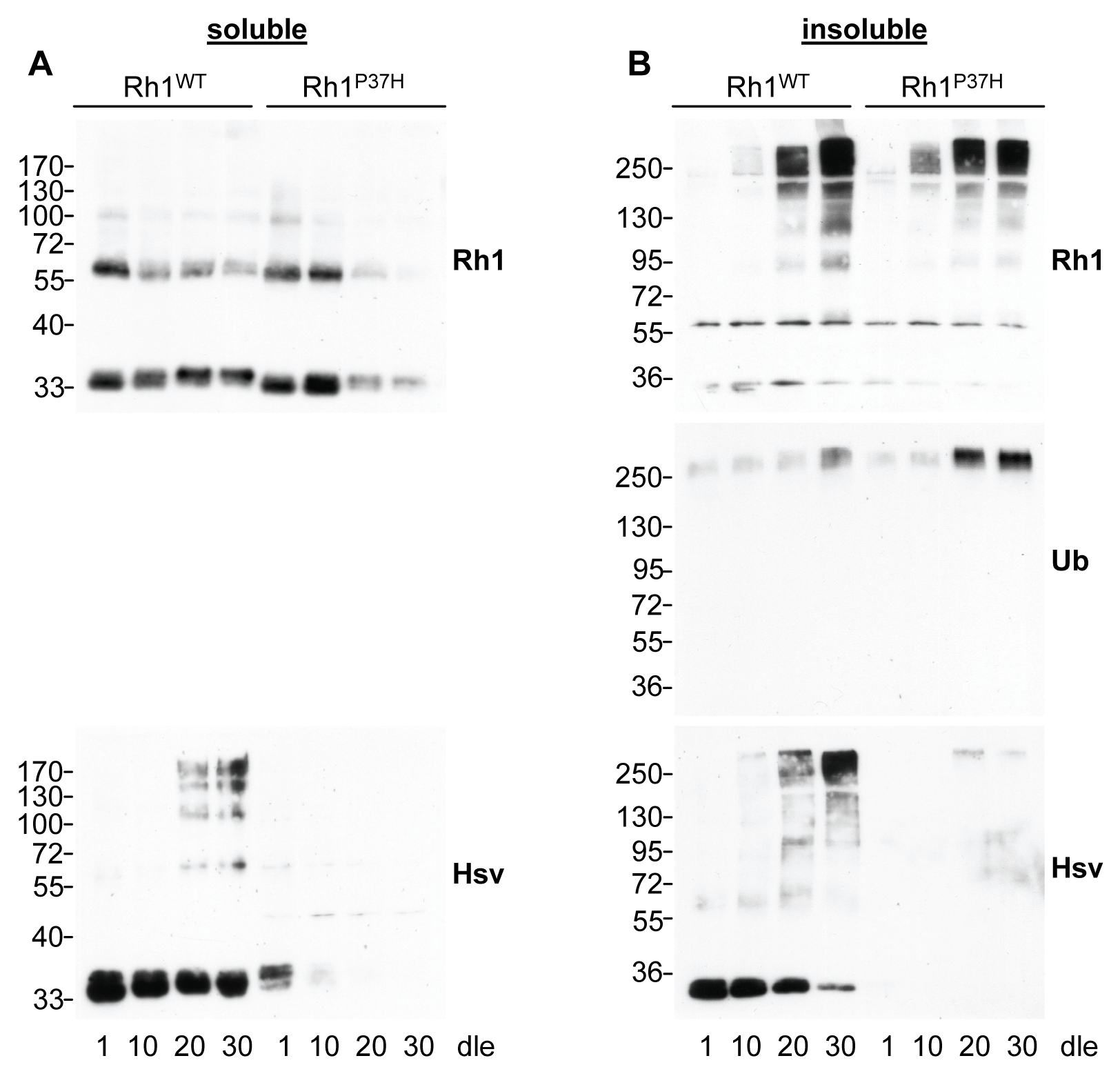

Supplement: Figure S4 — Loss of Rh1P37H in Rh1P37H-expressing flies exposed to light. (A,B) Immunoblots revealing the abundance of total (endogenous and ectopic, Rh1 antibody) and ectopic (hsv antibody) Rh1 in detergent-soluble (A) or insoluble (B) fractions obtained from retinas of Rh1WT;Rh1+/+ or Rh1P37H;Rh1+/+ flies exposed to light for the indicated durations. Aggregates were independently labeled with an Ubiquitin-specific antibody (B). (1.11 MB TIF) [file pgen.1001075.s004.tif]

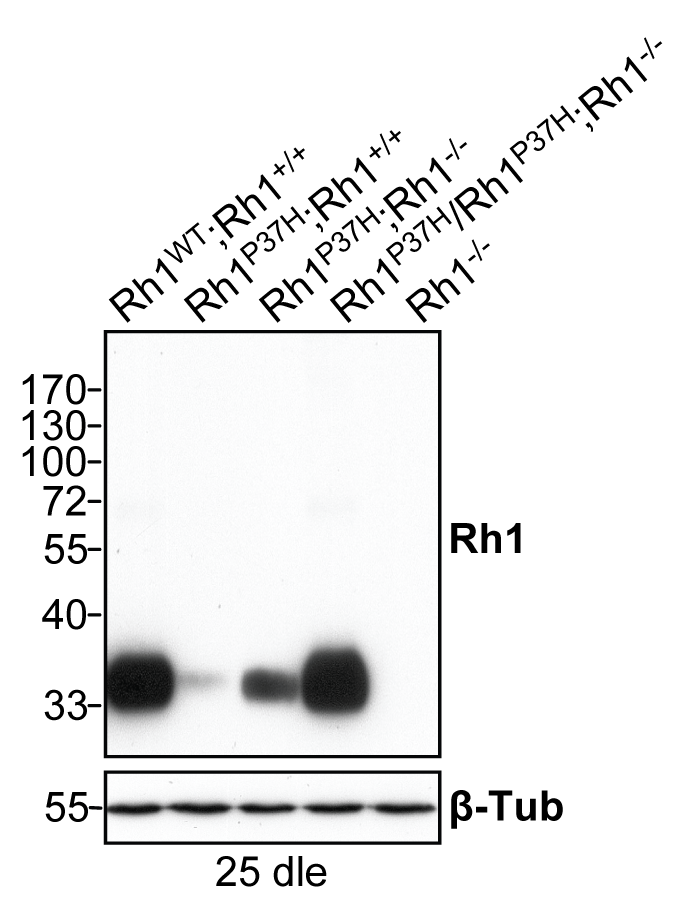

Supplement: Figure S5 — Rescue of retinal degeneration in Rh1P37H-expressing flies lacking endogenous Rh1. Immunoblot showing the levels of total Rh1 (Rh1 antibody) in detergent-soluble fractions obtained from retinas of flies of indicated genotypes reared at light for 25 days. Rh1P37H transgene expression in a Rh1 null background (lanes 3 and 4) suppresses retinal degeneration and prevents the loss of Rh1 seen in Rh1P37H;Rh1+/+ flies (lane 2). β-Tubulin (β-Tub) served as loading control. (0.27 MB TIF) [file pgen.1001075.s005.tif]

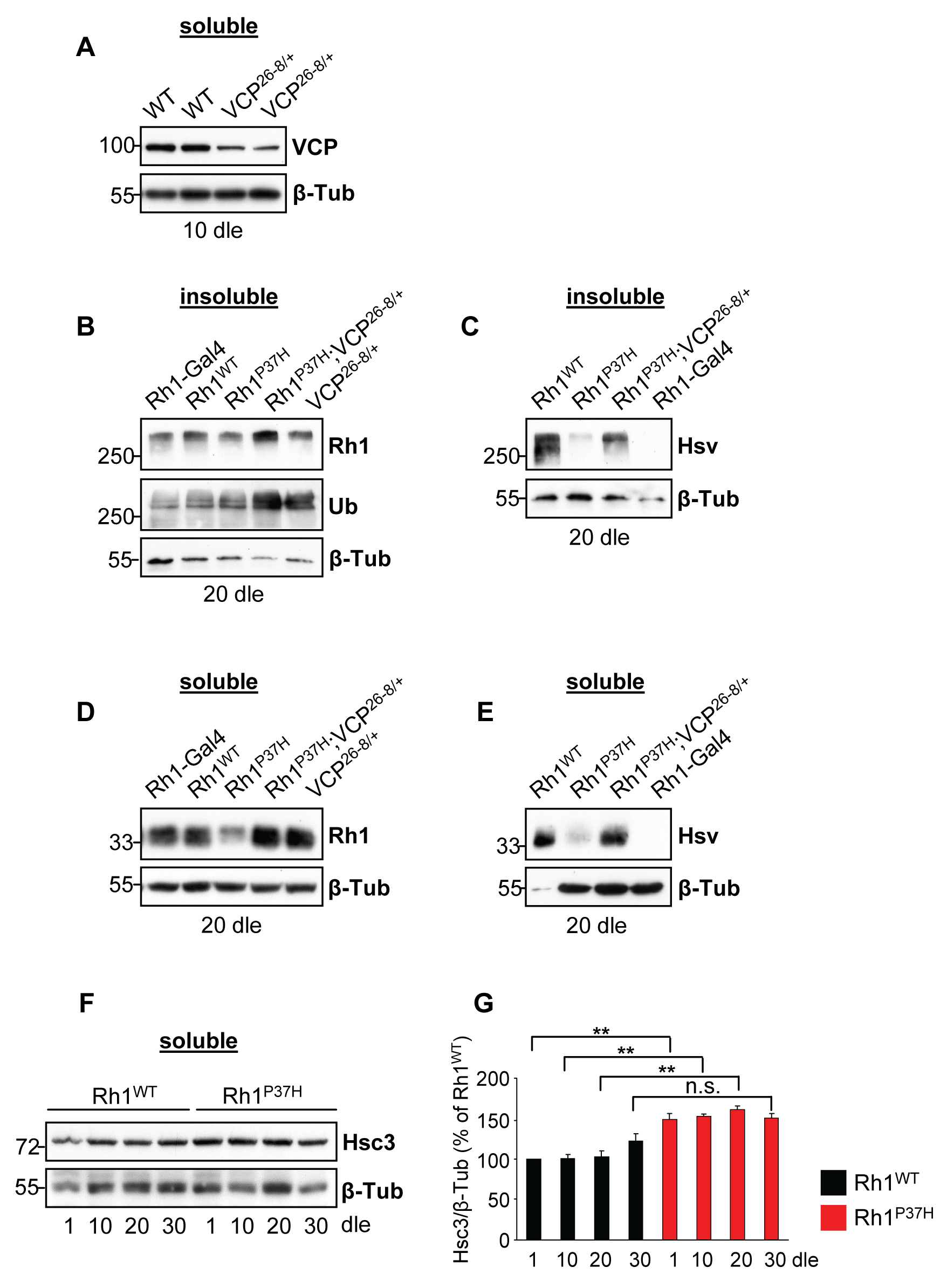

Supplement: Figure S6 — VCP inactivation increases Rh1 aggregate load and restores the levels of mature Rh1 in Rh1P37H-expressing flies. (A) Flies carrying the VCP hypomorphic allele VCP26-8 have reduced levels of VCP. Adult WT and VCP26-8/+ (genotype: VCP26-8/+;Rh1+/+) flies were tested for VCP expression levels using a VCP-specific antibody. VCP levels are significantly reduced in VCP26-8/+ flies, as compared to WT flies. (B,C) Immunoblots showing levels of total (endogenous and ectopic, Rh1 antibody; B) and ectopic (hsv antibody; C) Rh1 aggregates in flies of indicated genotypes after 20 days of light exposure (dle). An Ubiquitin-specific antibody was used to independently label aggregates. The amount of aggregated (endogenous and ectopic) Rh1 increases in Rh1P37H;VCP26-8/+;Rh1+/+ flies compared to control and mutant Rh1P37H;Rh1+/+ flies (B). Aggregates containing the ectopic Rh1P37H are also rescued from degradation after partial VCP inactivation (C). (D,E) Immunoblots revealing the levels of total (endogenous and ectopic, Rh1 antibody; D) and ectopic (hsv antibody; E) mature Rh1 in flies of indicated genotypes after 20 dle. There is loss of mature Rh1 in Rh1P37H;Rh1+/+ flies, which is rescued when reducing VCP function (D). Loss of ectopic mature Rh1P37H in Rh1P37H;Rh1+/+ flies is also rescued in Rh1P37H;VCP26-8/+;Rh1+/+ flies (E). Ectopic Rh1 was hsv-tagged in Rh1WT;Rh1+/+ or Rh1P37H;Rh1+/+ flies; no hsv signal was detected in Rh1-Gal4 flies which lacked hsv-tagged Rh1 (E). Please note that 10-fold less protein was loaded for Rh1WT;Rh1+/+ flies (E). (F) Immunoblot revealing the levels of Hsc3 in Rh1WT;Rh1+/+ and Rh1P37H;Rh1+/+ retinas, exposed to light for increasing durations. A 1.5-fold increase of Hsc3 levels is seen in Rh1P37H;Rh1+/+ versus Rh1WT;Rh1+/+ retinas starting at day 1. β-Tubulin (β-Tub) served as loading control. (G) Quantification of Hsc3 expression levels. The results are expressed as mean percentage compared to Hsc3 levels in Rh1WT;Rh1+/+ retinas at day 1 (100%) and [file pgen.1001075.s006.tif]

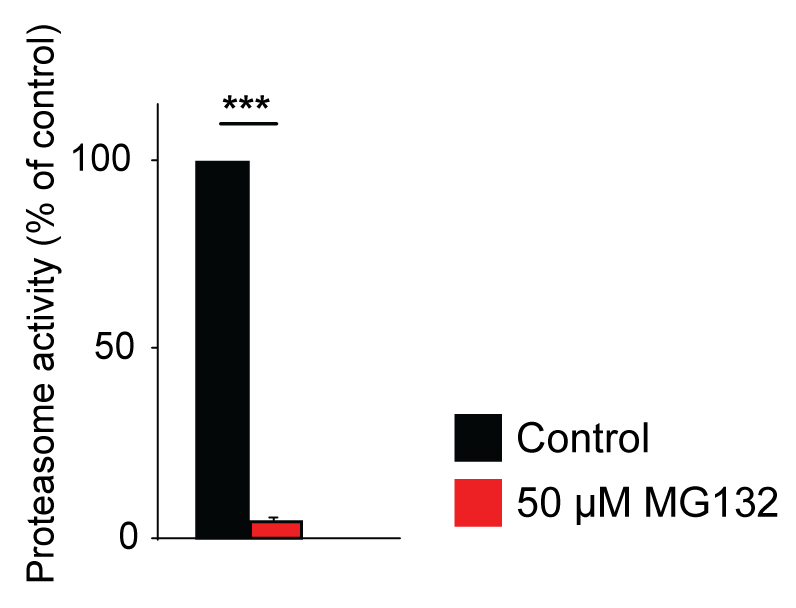

Supplement: Figure S7 — The proteasome inhibitor MG132 potently suppresses proteasome activity in Drosophila. Head fly lysates were assayed for the proteasome activity in the absence (control, 0.5% DMSO) or presence of the proteasome inhibitor MG132 (50 µM MG132 in 0.5% DMSO). The results are shown as mean percentage compared to proteasome activity levels in fly lysates treated with DMSO (set as 100%) and were averaged from three independent experiments (*** p<0.001 t-test). (0.05 MB TIF) [file pgen.1001075.s007.tif]

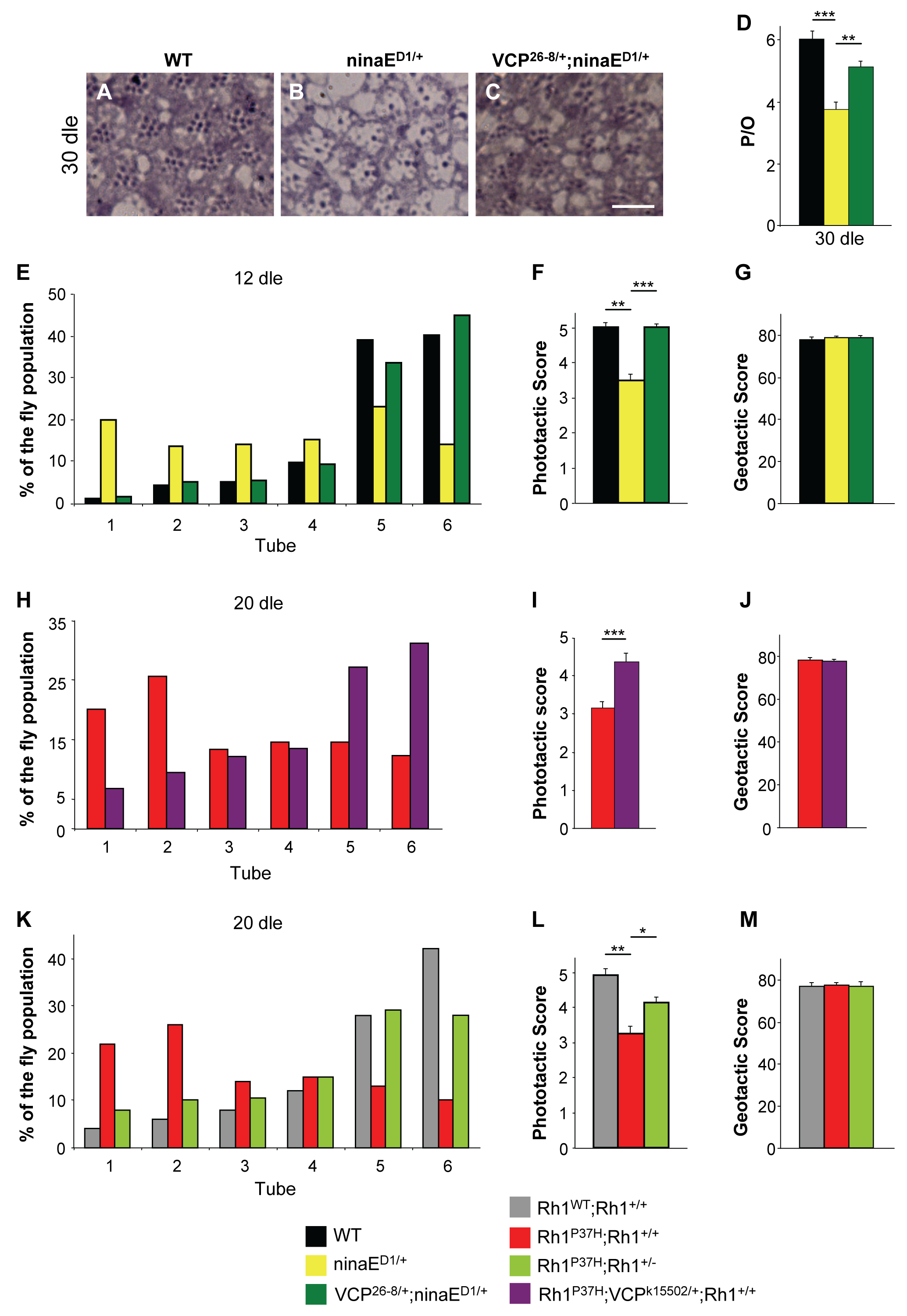

Supplement: Figure S8 — Retinal pathology in ninaED1 and Rh1P37H flies is rescued by VCP or Rh1 inactivation. (A–C) Photomicrographs of toluidine blue-stained semithin eye sections of WT (A), ninaED1/+ (B), VCP26-8/+;ninaED1/+ (C) flies after 30 days of light exposure (dle). Scale bar is 50 µm. (D) Quantification of average number of photoreceptors/ommatidium (P/O) (n>6 animals/group, ** p<0.01 and *** p<0.001 t-test). Decreasing VCP function suppresses retinal degeneration caused by ninaED1. (E) Phototaxis histogram after 12 dle revealing the light response of flies of indicated genotypes. ninaED1/+ flies show visual impairment relative to WT flies, while VCP26-8/+;ninaED1/+ flies display rescue of visual acuity. Between 250–300 flies were scored/genotype. (F) Phototactic score (PS) of WT, ninaED1/+ and VCP26-8/+;ninaED1/+ flies after 12 dle (n = 250–300 flies/group, ** p<0.01 and *** p<0.001 t-test). (G) Geotactic score of WT, ninaED1/+ and VCP26-8/+;ninaED1/+ flies after 12 dle. Flies from all genotypes display similar geotactic scores. (H) Phototaxis histogram after 20 dle revealing the light response of flies of indicated genotypes. While Rh1P37H;Rh1+/+ flies show visual impairment, Rh1P37H;VCPk15502/+;Rh1+/+ flies have an improved visual acuity. Between 150–200 flies were scored/genotype. (I) PS of Rh1P37H;Rh1+/+ and Rh1P37H;VCPk15502/+;Rh1+/+ flies after 20 dle (n = 150–200 flies/group, *** p<0.001 t-test). (J) Geotactic score of Rh1P37H;Rh1+/+ and Rh1P37H;VCPk15502/+;Rh1+/+ flies after 20 dle. Flies from both genotypes show similar geotactic scores. (K) Phototaxis histogram after 20 dle revealing the light response of flies of indicated genotypes. Rh1P37H;Rh1+/+ flies display visual impairment compared to Rh1WT;Rh1+/+ flies, while Rh1P37H;Rh1+/− flies have an improved visual acuity. Between 200–230 flies were scored/genotype. (L) PS of Rh1WT;Rh1+/+, Rh1P37H;Rh1+/+ and Rh1P37H;Rh1+/− flies after 20 dle (n = 200–230 flies/group, * p<0.05; ** p<0.01 t-test). (M) Geotactic score of Rh1 [file pgen.1001075.s008.tif]
