# Supplementary material for: Inactivation of VCP/ter94 Suppresses Retinal Pathology Caused by Misfolded Rhodopsin in Drosophila
Source: PLoS Genet. 2010 Aug 26;6(8):e1001075. doi: 10.1371/journal.pgen.1001075 (PMC2928793; doi:10.1371/journal.pgen.1001075)
Supplement: Text S1 — Supplementary Materials and Methods. Measurement of proteasome activity from fly head extracts. (0.03 MB DOC) [file pgen.1001075.s009.doc]

**Supplementary Materials and Methods**

**Measurement of proteasome activity**

Proteasome peptidase activity in *Drosophila* lysates was assessed colorimetrically using a synthetic peptide substrate, succinyl-Leu-Leu-Val-Tyr-7-amino-4-methyl-coumarin (Suc-LLVY-AMC, Sigma-Aldrich) as previously described [S1]. Proteasome activity was measured in the presence and absence of the proteasome inhibitor MG132 (50 µM in 0.5 % DMSO, Sigma-Aldrich). In order to preserve the proteasome activity, freshly collected control *Drosophila* heads were gently homogenized in buffer B (25 mM Tris-HCl pH 7.5, 2 mM ATP, 5 mM MgCl_2_ and 1 mM dithiothreitol) and incubated during 30 minutes on ice. Homogenates were centrifuged at 20.000 *g* for 20 minutes at 4˚C. Supernatants were then centrifuged at 20.000 *g* for 10 minutes at 4˚C and normalized for total protein using Protein Assay kit (Bio-Rad). A protein concentration of 0.3-0.5µg/µl is suitable for the assay. 10 µl of lysates were added in a 96 well-plate on ice. Then, 90 µl of assay buffer (100 mM Tris-HCl pH 8.0, 0.1 mM Suc-LLVY-AMC) supplemented with either 50 µM MG132 in 0.5 % DMSO or 0.5 % DMSO only was added in the dark and the plate was incubated at 37˚C during 1 hour in the dark. Fluorescence was red using a 380/460 nm filter set from a Synergy HT microplate reader.

**Supplementary Reference**

S1. Tonoki A, Kuranaga E, Tomioka T, Hamazaki J, Murata S et al. (2009) Genetic evidence linking age-dependent attenuation of the 26S proteasome with the aging process. Mol Cell Biol 29: 1095-1106.
